# Investigating the representation of uncertainty in neuronal circuits

**DOI:** 10.1371/journal.pcbi.1008138

**Published:** 2021-02-12

**Authors:** Guillaume P. Dehaene, Ruben Coen-Cagli, Alexandre Pouget

**Affiliations:** 1 University of Geneva, Département des neurosciences fondamentales, Geneva, Switzerland; 2 Albert Einstein College of Medicine, Bronx, Department of Systems & Computational Biology & Department of Neuroscience, New York, United States of America; 3 Gatsby Computational Neuroscience Unit, London, United Kingdom; Imperial College London, UNITED KINGDOM

## Abstract

Skilled behavior often displays signatures of Bayesian inference. In order for the brain to implement the required computations, neuronal activity must carry accurate information about the uncertainty of sensory inputs. Two major approaches have been proposed to study neuronal representations of uncertainty. The first one, the Bayesian decoding approach, aims primarily at decoding the posterior probability distribution of the stimulus from population activity using Bayes’ rule, and indirectly yields uncertainty estimates as a by-product. The second one, which we call the correlational approach, searches for specific features of neuronal activity (such as tuning-curve width and maximum firing-rate) which correlate with uncertainty. To compare these two approaches, we derived a new normative model of sound source localization by Interaural Time Difference (ITD), that reproduces a wealth of behavioral and neural observations. We found that several features of neuronal activity correlated with uncertainty on average, but none provided an accurate estimate of uncertainty on a trial-by-trial basis, indicating that the correlational approach may not reliably identify which aspects of neuronal responses represent uncertainty. In contrast, the Bayesian decoding approach reveals that the activity pattern of the entire population was required to reconstruct the trial-to-trial posterior distribution with Bayes’ rule. These results suggest that uncertainty is unlikely to be represented in a single feature of neuronal activity, and highlight the importance of using a Bayesian decoding approach when exploring the neural basis of uncertainty.

## Author summary

In order to optimize their behavior, animals must continuously represent the uncertainty associated with their beliefs. Understanding the neural code for this uncertainty is a pressing and critical issue in neuroscience. Following a long tradition, some studies have investigated this code by measuring how average statistics of neural responses (like the tuning curves) correlate with uncertainty as stimulus characteristics are varied. We show that this approach can be very misleading. An alternative consists in decoding the neuronal responses to recover the posterior distribution over the encoded sensory variables and using the variance of this distribution as the measure of uncertainty. We demonstrate that this decoding approach can indeed avoid the pitfalls of the traditional approach, while leading to more accurate estimates of uncertainty.

## Introduction

Animal behavior can adapt efficiently in the face of uncertainty. For example, when sensory stimuli are ambiguous, behavior is more variable and biased towards prior expectations than for informative stimuli [1]. Therefore, neuronal activity must continuously represent how certain an animal should be about its beliefs, that is, at any given time, the response of neurons must represent not only an estimate of the encoded variables, such as the direction of motion of objects, or their color, but also the uncertainty around these estimates. How is this momentary uncertainty represented? Answering this question is fundamental to understanding how the brain is able to implement or approximate statistical inference. Indeed, knowing the momentary uncertainty is critical to performing optimal multisensory integration, marginalization of nuisance variables and, more generally, all basic operations of Bayesian inference [2,3]. The specific format in which the uncertainty is represented thus constrains how these key operations can be implemented in the brain.

Two main approaches have been suggested to determine how uncertainty is represented in various brain regions. A first possibility consists in taking the perspective of a downstream area that has to extract the information from upstream neuronal activity, which we term the ‘Bayesian decoding’ approach. This approach starts from the so-called Bayesian ideal observer, whose goal is to compute the posterior probability distribution of the possible values of a sensory stimulus given the observed sensory inputs [1], namely *p*(stimulus | sensory input). According to this view, the activity of populations of sensory neurons should represent this posterior, which can then be used to perform Bayesian inference [2,4,5]. Note that what we call Bayesian decoding approach goes beyond previous applications of decoding methods that decode only a stimulus value or an animal’s decision, as opposed to the full probability distribution [6,7].

**Fig 1:**
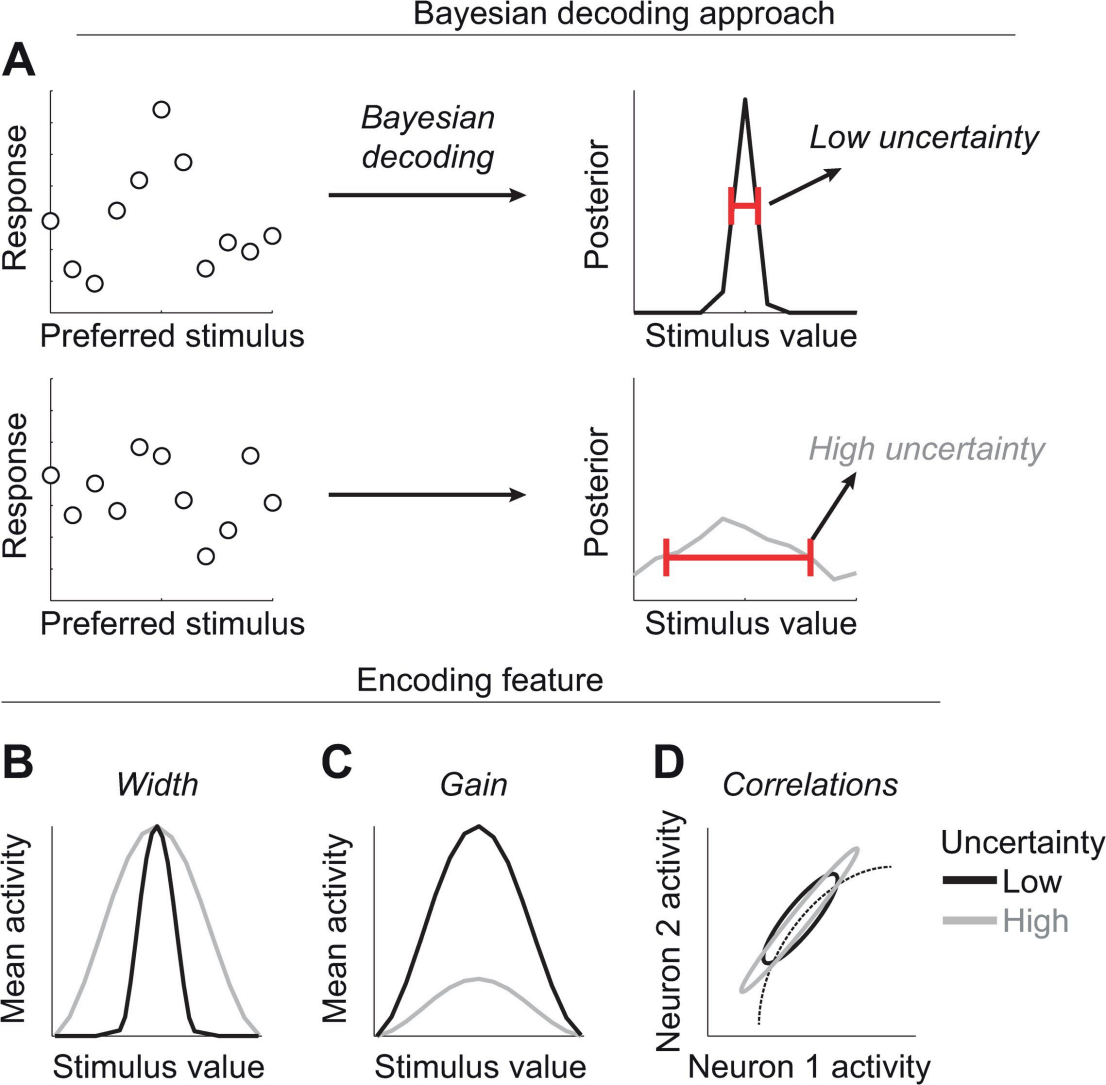
Correlational and Bayesian decoding approaches to uncertainty. (A) In the Bayesian decoding approach, the posterior distribution over the stimulus values (right panels) is decoded from the full pattern of neuronal activity in the population (left panels: each circle denotes the spike count of a neuron with preferred stimulus indicated on the abscissa). This yields both an estimate of the stimulus and a measure of the uncertainty of this estimate. Uncertainty is defined operationally as the variance of such posterior (red bars in the right panels). (B-D) In the correlational approach, it is assumed that a specific feature of neuronal activity, such as tuning curve width (B) or gain (C), or noise correlations (D) encode sensory uncertainty. It is then possible to identify such a code by observing which features of neuronal activity correlate with the information content of the stimulus.

Although the Bayesian decoding approach does not specify the form of the neuronal representation of the distribution, several possibilities have been considered with different implications for the form of the decoder. For instance, uncertainty might be encoded in the gain or amplitude of the tuning curves, which is a specific instance of the more general theory of linear Probabilistic Population Codes [8] (lPPC), according to which the logarithm of the distribution can be decoded simply by taking linear combinations of neuronal activity. Therefore, the decoding approach could be used to test experimentally specific models for the representation of momentary uncertainty, by decoding the distribution from the activity of sensory neurons using the model under consideration, estimating its uncertainty (Fig 1A), and comparing this estimate to the ideal observer uncertainty as well as to the experimentally measured behavioral uncertainty. The difficulty in testing this approach experimentally is that it requires simultaneous recordings from a large population of neurons, which is not yet feasible in many instances [9], although there is some initial evidence supporting this approach [10].

A second approach to studying the representation of uncertainty builds on the traditional definition of tuning curves, and has also the advantage that it can be applied to single-neuron recordings. Just like tuning curves are constructed by changing the value of a stimulus and measuring the corresponding changes in the mean response of a neuron, the representation of uncertainty has often been investigated by manipulating the information content of the stimulus while monitoring changes in specific features of neuronal responses. We will refer to this as the correlational approach.

This approach is appealing because, under different theories, different features can be related to uncertainty. For example, one could use the width of the tuning curves by setting the width proportional to the uncertainty [11] (Fig 1B). The gain or amplitude of the tuning curve could also play a role by being inversely proportional to uncertainty [8] (Fig 1C). Yet another possibility would be to make use of noise correlations [9] (Fig 1D). Indeed, under the right assumptions, uncertainty can be proportional to the overall level of noise correlations [12]. Lastly, under the hypothesis that neuronal activity represents samples from the target probability distribution [13], uncertainty would be reflected in the variance of the neuronal activity over an averaging time window. Following this correlational approach, Cazettes et al [14] recently found that, in the inferior colliculus (IC) of the barn owl, the uncertainty over the location of a sound source correlates with the width of the tuning curves better than with their gain, and suggested that therefore tuning width may encode uncertainty. Other studies are also suggesting that the width of place fields in the hippocampus encodes the uncertainty about the location of the animal [15], while in premotor cortex the width of the tuning curves to movement direction encodes the uncertainty in arm movement [16].

A key limitation of the correlational approach is that it has so far been only applied to identify correlates of the information content of the stimulus, which is related to the uncertainty averaged across trials, as opposed to the trial-to-trial, or momentary, uncertainty experienced by the animal. Indeed, this approach seeks correlations between the information content and features of single neuron responses averaged across trials. For instance, in the study of Cazettes et al, the average uncertainty of the location of an auditory stimulus is controlled by a quantity known as the binaural correlation, or BC, a measure proportional to information. They varied the BC across blocks of trials and reported that the width of the tuning curves—obtained by averaging activity within a block—varies with BC across blocks. However, the uncertainty experienced by the subject can vary even when the BC is fixed from trial-to-trial due e.g. to variations in the auditory stimulus, noise in the cochlea or fluctuations in the attentional level of the animal. These trial-by-trial variations are critical for behavior and must be represented in neuronal activity. One might imagine that features that correlate with average uncertainty also correlate well with momentary uncertainty, but this is not necessarily the case as we will show in this study. In order to assess whether the correlational approach is successful, it is essential to estimate the momentary uncertainty from the feature(s) identified by the correlational approach, which, to our knowledge, has never been performed on actual data.

To illustrate this, we compare these alternative approaches in a novel, biologically realistic model of sound localization based on Interaural Time Difference (ITD): the difference in the time it takes for a sound to reach the two ears. Our model implements a close approximation to the Bayes-optimal solution to the localization task in the presence of noise in the sound [11,14,17]. We show that that this model captures known features of the auditory pathway physiology in both the IC and OT, as well as behavioral data, and confirmed that momentary uncertainty can be accurately estimated by applying Bayesian decoding to the trial-by-trial neuronal activity.

In contrast, we report that the correlational approach can be greatly misleading: features such as width of tuning curves, can be highly correlated with average uncertainty, while providing very poor estimates of momentary uncertainty. These results also suggest that momentary uncertainty is unlikely to be encoded in a single feature of neuronal activity.

## Results

### Ideal observer for auditory localization accounts for behavioral bias and variability

**Fig 2:**
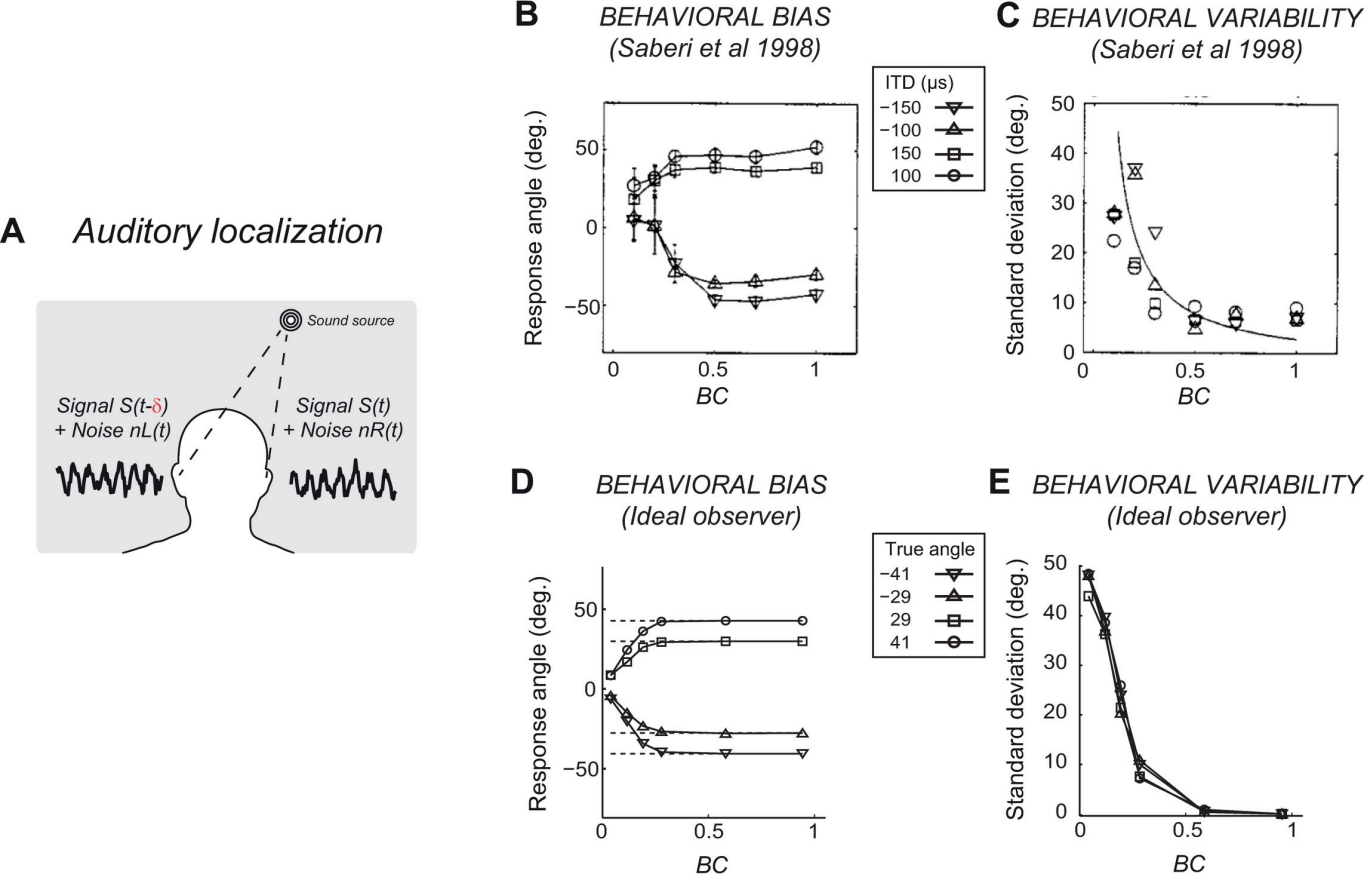
Ideal observer reproduces owl localization behavior. (A) In the auditory localization task, the azimuth of the sound source has to be estimated using the ITD (denoted by δ). (B) Barn owl head-turning responses at different levels of BC. Each curve corresponds to a different value of the true ITD. At low BC, head turns are on average biased towards the front (0 deg). (C) Variability of the head-turning responses. Different symbols correspond to different true ITDs. The continuous line is an exponential fit. At low BC, responses are more variable. (B,C) Replotted from Saberi et al (1998). Angles are measured with a precision of 4°. (D,E) Same as (B,C) but for the post-marginalization ideal observer. The true azimuth (values reported in the inset) is indicated by the dashed lines in (D).

We considered a specific behavioral task: localizing a sound source based on the ITD, that is, the time delay between the arrival of the same sound to the left and right ears (Fig 2A; see Methods Section “Uncertainty in the auditory localization task” for details). The objective is for the observer to estimate the ITD, namely the offset in time of the signal in the left ear. Following classical experiments by Saberi et al. [17], we generated sounds with one common white-noise process (the “signal”), plus independent white noise added to each ear (the “noise”). Mathematically, denoting the signal in the right and left hears as *s*_*R*_,*s*_*L*_, we have:

$$\left\{ \begin{array}{l} s_{R}(t)=\sigma_{S}s(t)+\sigma_{N}\eta_{R}(t)+\sigma_{0}\nu_{R}(t)\\ s_{L}(t)=\sigma_{S}s(t-\delta )+\sigma_{N}\eta_{L}(t)+\sigma_{0}\nu_{L}(t) \end{array}\right.$$ 1

The two signals are offset by the ITD *δ*. *s*(*t*),*η*_*R*_(*t*),*η*_*L*_(*t*),*ν*_*R*_(*t*),*ν*_*L*_(*t*) represent five independent white noise vectors. The parameters *σ*_*S*_ and *σ*_*N*_ control the relative size of the signal and the noise. We further added another independent noise with fixed amplitude *σ*_0_ = 0.9, to model internal noise which might be introduced, for example, at the level of hair cells in the cochlea. This additional noise was necessary for the model to fit behavioral data. We controlled the information content of the stimuli, and thus indirectly the average uncertainty about ITD, by varying the relative size of the signal and external noise components. This is quantified by the correlation between the sounds reaching each ear, or Binaural Correlation (BC; Eq 2; also known as Interaural Correlation).

$$BC=\frac{\sigma_{S}^{2}}{\sigma_{S}^{2}+\sigma_{N}^{2}}$$ 2

Notice that the BC only depends on the external noise values, controlled by the experimenter, and does not take into account the internal noise. A value of BC close to 1 indicates that the external noise is small relative to the signal and that estimation of the ITD is only limited by the internal sensory noise; conversely, BC equal to 0 means there is no information about ITD. We will investigate the momentary uncertainty by assuming that it is identical to that of an ideal observer which we describe next. This might differ from the perceptual momentary uncertainty of animal subjects (see also Discussion). Perceptual momentary uncertainty has not been measured directly for auditory localization to our knowledge, but, as we show below, the ideal observer reproduces accurately other existing behavioral data.

The ideal observer computed the likelihood function of ITD given the sounds, and combined it with prior information to obtain a posterior distribution over ITD *p*(*δ*|**s**_*R*_,**s**_*L*_). The details of the derivation are given in Methods Sections “Pre-marginalization ideal observer for known *BC* level” and “Ideal observer with marginalization of the *BC* level”. Note that, given the finite size of the head, ITD can only take values in a limited range: therefore, we assumed that the prior distribution of the ideal observer takes the form of a uniform prior, i.e. flat over the allowed ITD range and zero outside (assuming instead a Gaussian prior [11] centered at 0 had little impact on our results; see S1 Text).

We started by considering the simple case for which BC is fixed and known to the subject. The ideal observer is then able to compute the posterior distribution of conditional on the value of BC *p*(*δ*|**s**_*R*_,**s**_*L*_,*BC*). In the following, we use the term “pre-marginalization” to refer to perfect knowledge of BC as opposed to the “post-marginalization” ideal observer, which we present in the next paragraph, which marginalizes out the unknown BC from the posterior distribution.

$$p\left(\delta |s_{R},s_{L}\right)=\int p\left(\delta |s_{R},s_{L},BC\right)p(BC)dBC$$ 3

In this case, it is straightforward to show that the pre-marginalization ideal observer should simply compute the cross-covariance between the signals at the two ears, at a range of possible ITDs, to obtain the log posterior distribution (Methods Section “Pre-marginalization ideal observer for known *BC* level”). The cross-covariance is a function of offset which measures the empirical covariance between **s**_*R*_(*t*) and **s**_*L*_(*t*+*δ*):

$$CC\left(\delta \right)=Cov\left(s_{R}(t),s_{L}(t+\delta )\right)$$ 4

Intuitively, this operation corresponds to evaluating the similarity between the right and left ear signals for different relative offsets. Note that, even for fixed BC, the empirical cross-covariance is random. The posterior width can thus vary on a trial-by-trial basis, causing the momentary uncertainty of the Bayesian ideal observer to fluctuate even in the absence of variation of the experimentally controlled information content.

However, in typical experiments, BC is varied randomly across trials and subjects are not informed about the specific value used on each trial [17]. Therefore, BC acts as a nuisance variable that the ideal observer has to marginalize out, as in Eq 3 (hence our terminology, pre- and post-marginalization). To our knowledge, no closed-form solution existed for this problem. We therefore developed a method to compute analytically a close approximation to the log posterior. The resulting post-marginalization ideal observer needs to compute the cross-covariance between the signals at the two ears *CC*(*δ*), divide it by a measure of their variance *V*, and apply a specific nonlinearity *h* (Methods Eq 24).

$$p\left(\delta |s_{R},s_{L}\right)=h\left(\frac{CC(\delta )}{V}\right)$$ 5

**Fig 3:**
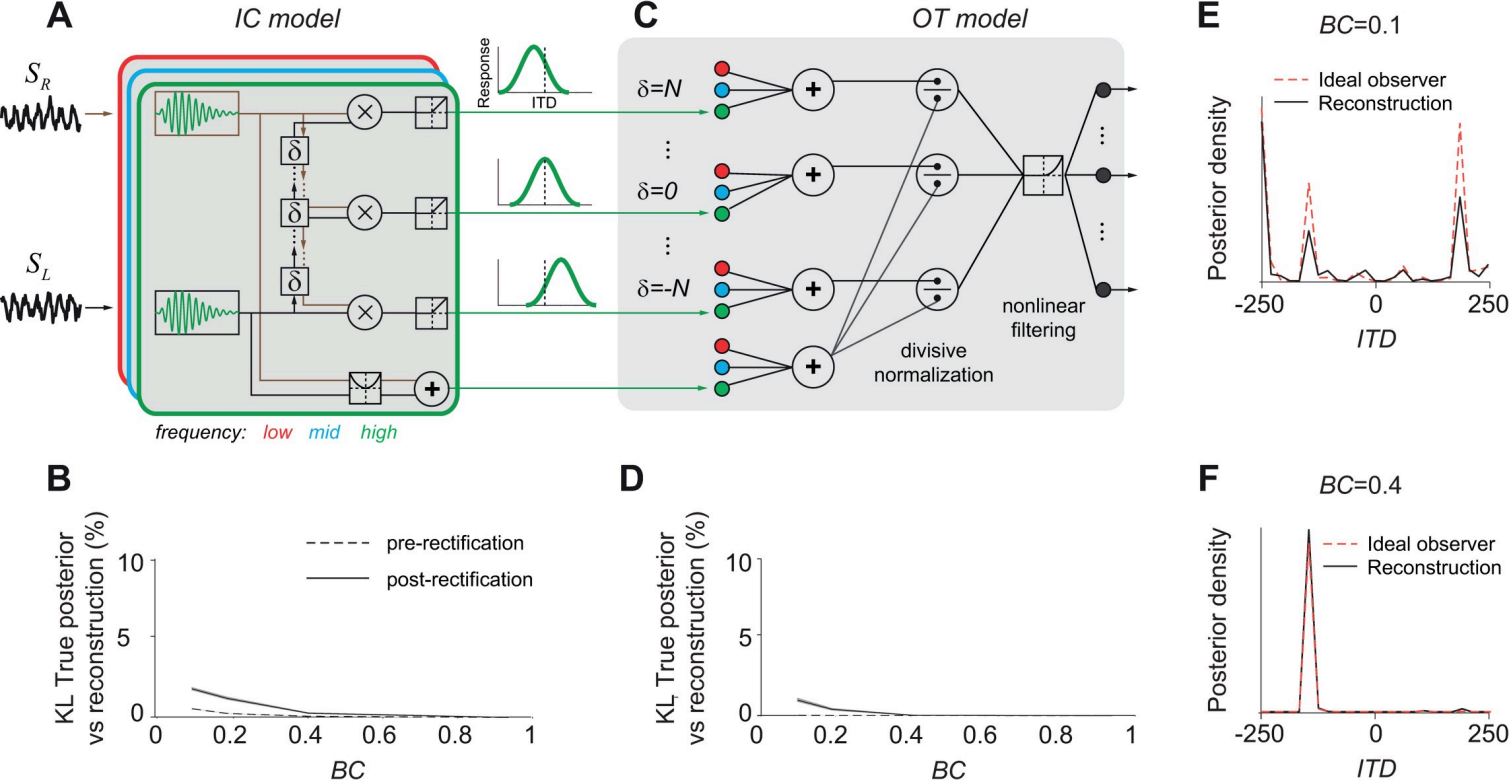
IC and OT models approximate the ideal observers. (A) Schematic model of IC. Sounds reaching the two ears are first convolved with bandpass filters, then delayed, then cross-correlated at several delays, and lastly rectified to obtain tuning curves to ITD. (B) Average KL divergence between the posterior distribution computed by the ideal observer and the posterior decoded from the model population activity before (dashed line) and after (continuous) rectification. KL was normalized to the KL between the ideal observer posterior and the priors. Shaded areas represent s.e.m. Note that the pre-rectification KL divergence differs from 0 purely due to the fact that we cannot exactly decode from a finite amount of training examples. (C) Schematic model of OT. Outputs of the IC model are first combined across frequency bands and divided by the signal energy. Then, the outputs are passed through a static nonlinearity, filtered and rectified; these operations are equivalent to a non-linear filtering stage. The color of the units indicates their frequency preference (low: red, mid: blue, high: green). (D) Same as (B) but for the OT model activity, after rectification. (E) Example posterior distribution in one trial with true ITD = -145 and BC = 0.1, for the post-marginalization ideal observer (dashed pink line) and the reconstruction from the output of the OT model population (continuous black). (F) Same as (E), but with BC = 0.4. Note that the small secondary peak around ITD = 200 is present only in the reconstruction, not in the ideal observer’s posterior.

We then asked whether the owl behavior in the localization task was consistent with the ideal observer. Saberi et al. [17] characterized the orienting behavior of barn owls presented with stimuli at four different ITDs with randomly interleaved BC. For small values of BC, the animal’s behavior exhibits a systematic bias towards central locations (Fig 2B) and becomes increasingly more variable (Fig 2C). We found that the post-marginalization ideal observer behaves in a qualitatively similar manner (Fig 2D and 2E; see S2 Text for comparison to the pre-marginalization case). Specifically, for each simulated trial we generated noisy signals to the right and left ears, then computed the log-posterior, and chose the ITD value that maximized the log-posterior to be the observer’s estimate (known as MAP, or maximum *a posteriori*, estimate; Methods Section “Ideal observer behavior”). At high BC, the log-posterior was narrowly peaked, with a MAP very close to the true ITD. Therefore, the behavior across trials showed very little variability. At lower BC however, the log-posterior was broader (compare the two example trials at low and high BC in Fig 3E and 3F, respectively) and its peak location could change substantially across trials, producing behavioral variability. The bias at low BC was simply a consequence of the uniform prior over the limited range of ITDs represented by the ideal observer. For instance, in the simulations of Fig 3, since the ITD range is [-250*μs*,250*μs*], if the true ITD were 150*μs*, an underestimation by 150*μs*, corresponding to a reported ITD of 0*μs*, is possible, whereas an overestimation error of 150*μs*, corresponding to a reported ITD of 300*μs*, would not be possible (see S3 Text). Note also that, for intermediate BC, the perceptual bias of the ideal observer is larger for large eccentricities of the true ITD (Fig 2D), consistent with the known stimulus-dependence of the perceptual bias [18]. We also note that the data show some deviations from our ideal observer: first, there is an asymmetry in the bias for left and right angles; this could be captured by changing the support of the uniform prior (see S3 Text), but could also reflect motor idiosyncrasies; second, the experimental variability does not plateau to zero at high BC, but it should be noted that angles were measured with a precision of 4 degrees.

A similar explanation for the bias was proposed by Fischer and Pena [11], who designed a neuronal model for which the population vector decoder closely approximates the MAP estimate. In such model, a Gaussian prior for central locations induced the bias. Another possible explanation for the bias is a cost proportional to head saccade amplitude, which could be easily incorporated in the proposed framework. Discriminating between these different explanations for the bias will require additional experiments explicitly designed for this purpose.

### Neuronal approximations of ideal observers match auditory pathway physiology

Given that the ideal observer reproduced behavioral data accurately, we asked how it could be implemented by a population of neurons (details in Methods Section “Neural implementations of the ideal observers”). Specifically, we asked which computations should be performed on the raw sensory inputs such that the true log-posterior over ITD can be reconstructed by a weighted sum of the neuronal activity across the population (Methods Eq 34). A population that satisfies this requirement is called a linear Probabilistic Population Code (lPPC [19]). We designed two populations organized hierarchically, to mimic processing in the IC and OT that approximated the ideal observers for known and unknown BC respectively.

For the case of a fixed and known BC, model neurons simply needed to compute the cross-covariance of the signals from the two ears at different time lags (Methods Section “Pre-marginalization ideal observer for known *BC* level”). This is equivalent to the traditional model of the early auditory pathway [20] (Fig 3A). The signals were first decomposed in narrow frequency bands at each ear, mimicking cochlear processing, yielding the filtered signals:

$$\begin{array}{l} {\tilde{s}}_{n,R}=F_{n}^{⊤}*\left(F_{n}^{⊤}*s_{R}\right)\\ {\tilde{s}}_{n,L}=F_{n}^{⊤}*(F_{n}^{⊤}*s_{L}). \end{array}$$ 6

where the subscript *n* refers to the frequency of the filter and the symbol * denotes the convolution operator. Next, frequency-specific cross-covariances were computed between the two filtered signals at a range of ITDs, producing the neuronal activity of a neuron tuned to a given ITD *δ* and frequency band *n*:

$$r_{n,\delta }=Cov({\tilde{s}}_{n,R}(t),{\tilde{s}}_{n,L}(t+\delta )).$$ 7

Such tuning has been indeed observed in the nuclei of IC of the barn owl [21] and in the Medial Superior Olive of mammals [22,23].

This model is such that the log-posterior of the pre-marginalization ideal observer can be reconstructed by linear combinations of the neuronal activities. Indeed, this log-posterior is proportional to the cross-covariance across all frequencies, which can be reconstructed by summing the frequency-specific cross-covariances (Methods Section “Neural implementations of the ideal observers”, Eqs 31–32):

$$LL(\delta )\propto Cov(s_{R}(t),s_{L}(t+\delta ))=\sum_{n=1}^{4}Cov({\tilde{s}}_{n,R}(t),{\tilde{s}}_{n,L}(t+\delta ))=\sum_{n=1}^{4}r_{n,\delta }$$ 8

This population thus implements the pre-marginalization ideal observer in lPPC format. Note that we have designed this population to only encode the log-likelihood of values of *δ* which have non-zero prior probability. It thus encodes the prior implicitly.

However, neuronal firing rates cannot take negative values, thus we introduced a rectifying nonlinearity. We considered half-rectification (but see S4 Text for other nonlinear functions), and evaluated how well the log-posterior could be reconstructed from a linear combination of neural activities. In practice, the reconstruction weights used in the linear combination have to be learned from experience (for a downstream area) or from experimental data (for an experimenter). In this study we used multiclass logistic regression, a classification method that assumes neuronal activity is an lPPC, and searches for the weights that provide the best classification on a training dataset (see Methods Section “Decoding uncertainty from population activity”). Specifically, we used, on each trial the neuronal population activity as predictor and the true log-posterior distribution as the target. Importantly, if the population is a lPPC, then linear decoding coincides with Bayes’ rule and reconstructs the full log-posterior distribution over classes. Note this use of multiclass logistic regression is different from the more traditional approach, in which the target is the Dirac delta distribution at one specific class value (see Methods “Decoding uncertainty from population activity”). In experimental applications, training should be performed with this more traditional approach using the true stimulus as the target instead of a distribution, and the assumption that all the information is present in non-linear features of the activity should be checked [24–27]. We did not use regularization because the training set was larger than the number of weights by a factor of 100 (Methods Section “Decoding uncertainty from population activity”), but we used cross-validation, i.e. we evaluated the reconstructed log-posterior on a separate set of trials. We quantified the reconstruction quality by the Kullback-Leibler divergence (KL), a natural measure of dissimilarity between probability distributions: KL is zero for two identical distributions, and increases for increasingly different distributions. We further normalized the KL by dividing it by the KL between the true posterior and the prior, and expressed the result as a percent information loss [24]. If the model population indeed implemented the ideal observer with a lPPC, and we had infinite data for training, then the percentage information loss should be 0. However, the percentage information loss was not exactly zero for the pre-rectification model at low BC values (Fig 3B dashed-line), even though the pre-rectification model is optimal. In this case, the minimal information loss (less than 1%) is purely the result of using training dataset with a finite size.

Adding the rectification only had a marginal impact on the performance of the model. Fig 3B (solid line) shows that the reconstruction was almost perfect for large values of BC, whereas its quality decreased at low BC values, where the effects of the rectifying nonlinearity became more prominent. Nonetheless, the cross-covariance model provided a good approximation of the ideal observer overall, with only 3% loss of information at extremely low BC (BC = 0.1, note that, as we have just seen, one third of this information loss is likely due to a finite data set as indicated by the dashed line).

So far, we have shown that the popular cross-covariance model of the early auditory pathway closely approximates the pre-marginalization ideal observer in a lPPC. However, this relied on the assumption that BC was known exactly. More precisely, knowledge of BC was required because a different set of reconstruction weights had to be learned for each BC. As explained earlier, in typical experiments BC is not known to the subject, and has to be marginalized out through a complex nonlinearity. Therefore, we built a neuronal population that implemented a close approximation of that non-linearity, followed by an additional rectification step. Because the log-posterior can be decoded from this population with the same decoding weights regardless of BC level, this population is said to implement an invariant lPPC, or i-lPPC[28]. We then compared its response properties to those of OT, an area that lies downstream of the IC and contains a multimodal map of space used by owls for orienting [29].

As we have described, the post-marginalization ideal observer requires computing the cross-covariance, normalizing it and applying a specific non-linearity. These operations can be implemented in a neuronal model. As illustrated in Fig 3C, our model involved, first, frequency convergence, to compute the frequency-independent cross-covariance of *s*_*R*_,*s*_*L*_; second, divisive normalization to obtain the cross-correlation; third, the static nonlinearity (Methods Eq 24). Lastly, we included a linear filtering stage to obtain a distributed code, and a rectifying nonlinearity to produce the spike count of the OT model neurons. This model closely approximated the ideal observer with BC marginalization as expected (Fig 3D and 3F). Similar to the pre-marginalization case, the larger information loss (1%) at low BC was due mainly to the last rectification step, and partly because we used a finite data set.

**Fig 4:**
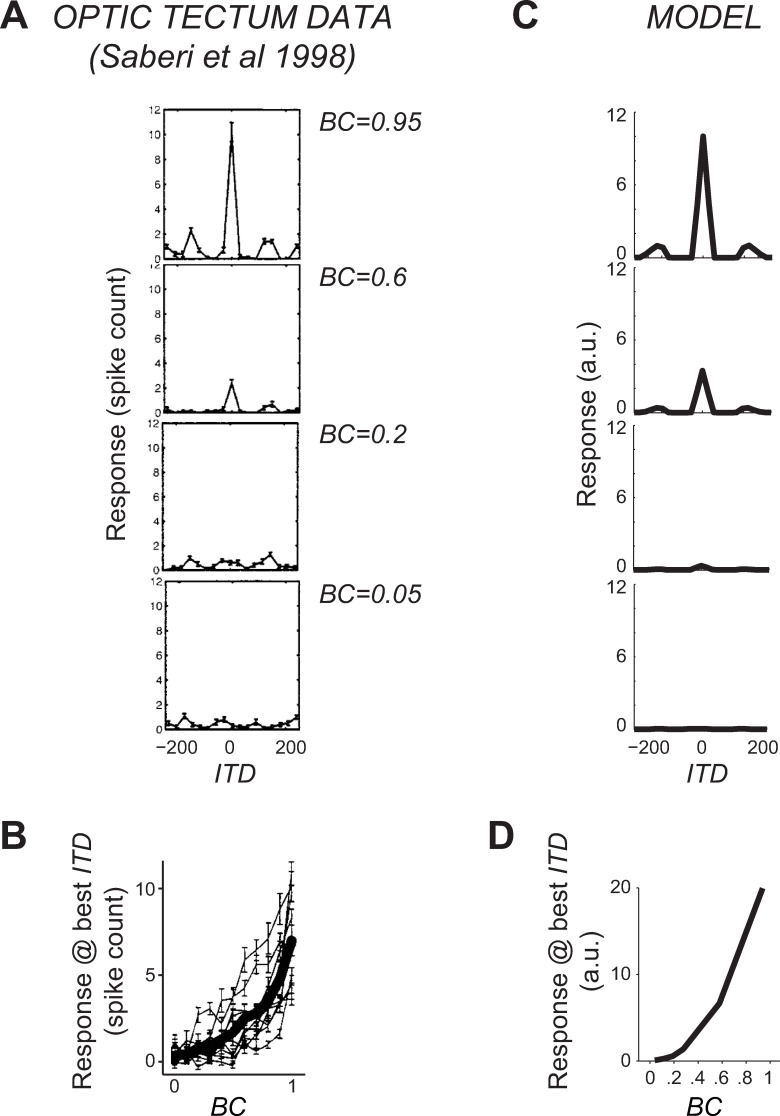
Comparison of OT model and data. (A) Tuning curve for ITD of a neuron recorded in owl’s OT. Each panel corresponds to a different BC. (B) Peak response as a function of BC. Thin lines represent individual neurons, thick line is the population average. Data replotted from Saberi et al.. (C,D) Same as (A,B) but for a model neuron.

We found that the model neuronal tuning curves for ITD qualitatively matched those observed in OT of barn owls [17], exhibiting a main peak at the preferred ITD and smaller side peaks (Fig 4A and 4B). Furthermore, when BC was decreased, the height of the peak decreased in the model in a way consistent with the physiology (Fig 4C and 4D). Although our final step of filtering the log-posterior to obtain a distributed code has several degrees of freedom, we found that a basic smoothing yielded an excellent qualitative match with the data. Therefore, we did not optimize this step any further, even though these free parameters can be tuned to improve this fit. The only notable free parameter is the level of internal sensory noise in Eq 1, which we kept fixed to the same value for both behavior (Fig 2) and neuronal activity (Fig 4) fits. The tuning curve shape and dependence on BC are largely independent of this parameter: it mostly affects the relative height of the central and side peaks. Note also that we have not tried to capture in detail response variability and covariability in IC or OT since very little experimental data is available for these quantities (but see [30]) and in particular for the fraction of this variability that strongly limits information [9]. Crucially however, noise in the sensory inputs induces response variability in our model, and such variability is shared between neurons, thus inducing so-called differential noise correlations [9] as we have shown previously for visual orientation processing [31]. Such differential correlations are the primary source of uncertainty, but current experimental data do not allow to estimate them in vivo.

In summary, we have shown that neuronal responses in IC and OT are qualitatively well reproduced by neuronal models that closely approximate lPPCs for known and unknown BC, respectively. Given that this approach based on ideal observer models provided a unified account of behavior and neuronal activity in the auditory localization task, we next asked what can be learned from it about the neuronal representation of momentary uncertainty.

### Estimating momentary uncertainty from neuronal activity

In the previous section we have shown that the true posterior distribution over ITD can be decoded accurately from neuronal activity in the models on a trial-by-trial basis. Here we focus instead on a specific aspect of the posterior distribution, namely its variance. This has proven to be a popular choice in the past literature [2,32] because it is a natural measure of uncertainty and, for Gaussian distributions, it provides the optimal weights for cue combination (S8 Text). Another natural measure of uncertainty is the posterior entropy, which also quantifies posterior spread and is less susceptible to small secondary peaks in multimodal distributions. We verified that all the results shown below also hold using this alternative measure (S5 Text). Note that much previous work has often relied on Fisher information to relate neural activity to uncertainty. Here, however, we focus on momentary uncertainty, while Fisher information can only be measured on average across repetitions of the same stimulus.

**Fig 5:**
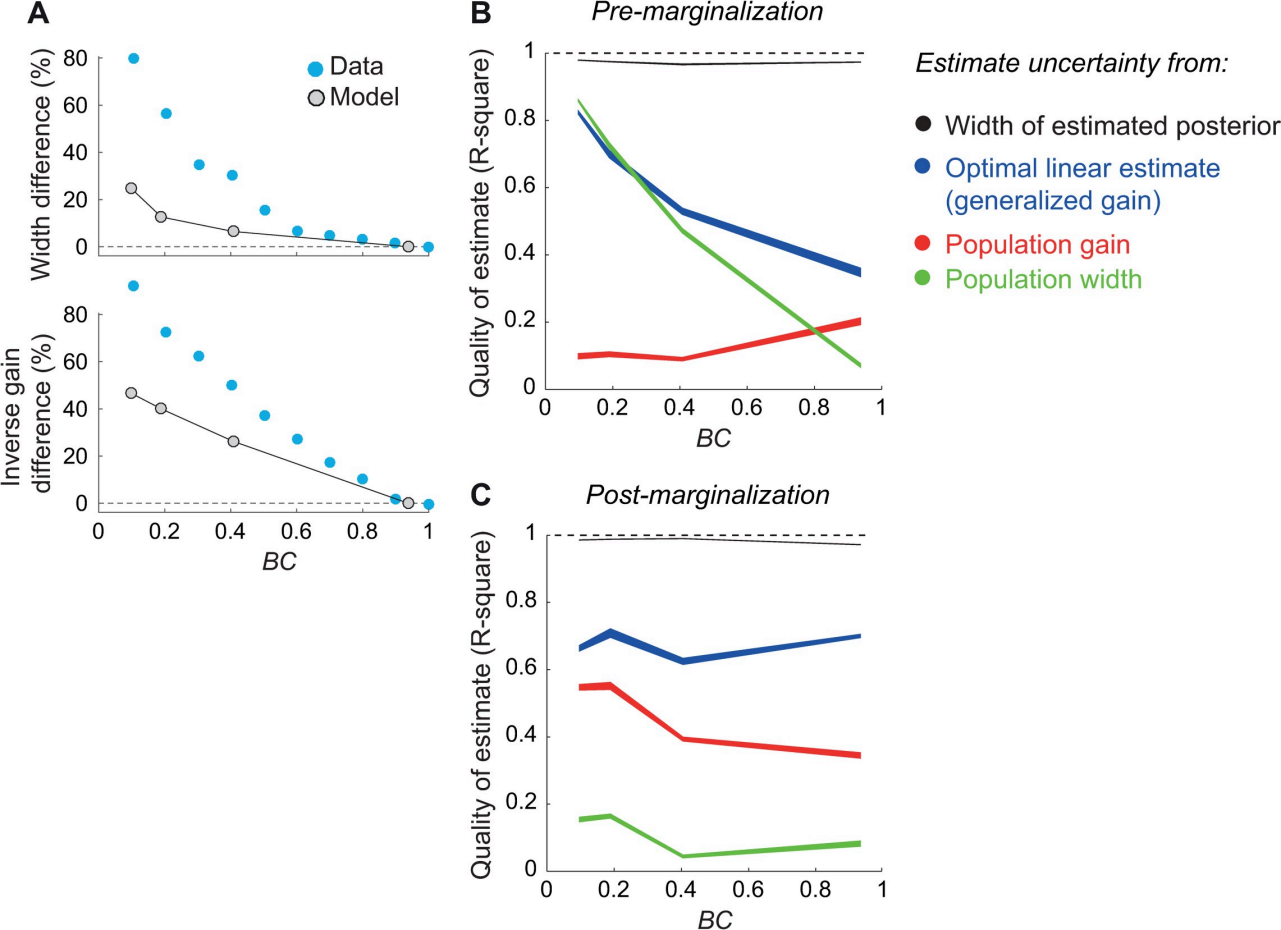
Uncertainty estimation quality from IC and OT neuronal activity. (A) Tuning cuve width and inverse gain as a function of BC, expressed as a percent change from the value measured at the highest BC. Gray symbols: models. Blue: data from Cazettes et al. [14]. (B) Performance of different estimators as a function of BC. The estimators used IC neuronal activity to estimate the uncertainty of the pre-marginalization ideal observer. Quality was measured by the cross-validated R^2^ between the ideal observer’s posterior log-variance and estimated log-variance. Black: uncertainty of the reconstructed posterior. Blue: uncertainty estimated by a linear fit to single neuron activity. Red: uncertainty estimated by a linear fit to the total neuronal activity. Green: uncertainty estimated by a linear fit of the width of population activity. Line width represents 95% c.i. (C) Same as (B), but using OT neuronal activity to estimate the uncertainty of the post-marginalization ideal observer. In this case performance is still split by BC, but the linear fits were performed by combining trials across all BC (i.e. the estimators, as well as the ideal observer, had no knowledge of the true BC value).

We first verified that the momentary uncertainty estimated from the decoded posterior reflected accurately the true momentary uncertainty of the ideal observer. We found that that is indeed the case: the variance of the approximate posterior estimated from the neural responses closely matched the variance of the posterior of the ideal observer (R^2^>0.95 for the log-variances at all BC levels, both pre- and post-marginalization; Fig 5, black). We then studied whether the correlational approach could be used to recover the trial-by-trial fluctuations of uncertainty. To this aim, we considered features of neuronal activity that have been shown to correlate with average stimulus information, and derived single-trial estimators of uncertainty that are consistent with the hypothesis that uncertainty is encoded in those features. More precisely, for each feature *f* we defined a scalar quantity *q*^*f*^(**r**) derived from single-trial population activity **r**, and then used linear regression to obtain an optimal linear estimator of the uncertainty of the ideal observer of the stimulus based on *q*^*f*^(**r**):

$$\log(v)=\beta_{0}+\sum_{f}\beta_{f}q^{f}\left(r\right)$$ 9

where *v* is the posterior variance of the ideal observer, and {*β*_*f*_} a set of coefficients on a training set comprising half of the trials. Estimation quality was measured by the cross-validated R^2^ computed on the other half of the data not used for training. The linear fit was optimized separately for every BC level for the IC model, and across all BC levels for the OT model. We first focused on the tuning curve width [14–16], and defined the following single-trial predictor (corresponding to the width of the population hill of activity):

$$\begin{array}{l} q^{w}(r)=\sum_{n}{\tilde{r}}_{n}{\left(\delta_{n}-\sum_{m}{\tilde{r}}_{m}\delta_{m}\right)}^{2}\\ \;{\tilde{r}}_{n}=\frac{r_{n}}{\sum_{m}r_{m}} \end{array}$$ 10

where *δ*_*n*_ is the preferred stimulus value of the *n*-th neuron. Second, we considered tuning curve gain [8], and defined the total activity across the population as predictor:

$$q^{g}(r)=\sum_{n}r_{n}$$ 11

We verified that both tuning curve width and gain correlated with BC (i.e. with average stimulus information; see S4 Text). For the IC model, we computed width and gain for different subpopulations corresponding to different preferred temporal frequencies, and used each subpopulation as a separate predictor, because simply summing the responses across frequency bands (as in models of ICx that include frequency-convergence) before computing the predictors reduced the quality of the estimates.

Lastly, we considered a generalization of gain, that uses each neuron’s activity as a predictor

$$q_{n}^{gg}(r)=r_{n}$$ 12

and therefore estimates uncertainty as a weighted sum of the population activity, with weights optimized to fit the uncertainty of the ideal observer. While generalized gain (Fig 5, blue lines) performed better than pure gain (Fig 5, red) or width (Fig 5, green), all features provided poor estimates. We verified that this was also true for other, nonlinear features that combined gain and width or that used neuronal correlations explicitly (S5 Text).

The particularly poor performance of the gain-based estimates might seem at odds with the fact that the log-posterior could be reconstructed nearly perfectly with a linear estimator (Fig 3) thus suggesting that the output layer of our model encodes ITD with lPPCs. Linear PPCs are sometime confused with codes in which uncertainty is proportional to the gain, but this is in fact not the case. In a lPPC, the log posterior distribution can be recovered via a linear combination of activity [19], but estimating the uncertainty, i.e., the width of the posterior distribution, may require a nonlinear operation. The uncertainty is linearly recoverable only if the weights used to recover the log posterior are quadratic functions of the encoded stimulus [19]. For instance, in Fig 5A, the performance of the optimal linear estimate decreases as BC increases because the weights are no longer a simple quadratic function of ITD.

Using a model of IC neurons similar to the one we used (i.e. frequency specific filtering, followed by cross-covariance and a static nonlinearity), Cazettes et al. [14] followed the correlational approach and reported that tuning-curve width was a major factor for uncertainty coding. However, they simply reported a correlation between the width of the tuning curves and average uncertainty. As our results show, this correlation does not imply that the behaviorally-relevant momentary uncertainty can be linearly estimated from the width of the population activity. However, another obvious difference between the two models is the choice of the static nonlinearity: half rectification in our case, versus exponential in Cazettes et al. [14] which provides a good fit to intracellular recordings in the external nucleus of IC. We therefore tested whether our conclusions where due to the specific non-linearity we used. In models with a sigmoid or exponential non-linearity, we found that reconstruction based on tuning curve width was as poor as for half-rectification (however, the sigmoid non-linearity might be a slightly improved fit to the physiology; see S4 Text). In such models, linear decoding of the posterior was furthermore impossible since these populations are not linear PPCs anymore. The approximate posterior obtained by linear decoding was thus completely incorrect, leading to bad uncertainty estimates. Therefore, our analysis illustrates the limitation of the correlational approach: even if a feature of neuronal activity correlates with average uncertainty, it might not capture momentary uncertainty accurately. We further address in Discussion the relevance of this observation for understanding perceptual behavior.

### Comparison to other models of OT and experimental predictions

The neuronal model we have proposed for OT, which assumes that this region represents the log-posterior of the post-marginalization ideal observer, requires a very specific nonlinearity, i.e, a form of divisive normalization (Fig 3C). To test the robustness of the approach, we first asked whether the log-posterior and its uncertainty could be accurately reconstructed using approximations to the exact nonlinearity, achieved by a cascade of simpler nonlinearities. We then compared new predictions of our model to previous descriptive models of OT.

We first verified that the precise implementation of the OT non-linearity was not critical. We trained a feedforward network with a simple rectifying non-linearity (a well-known universal approximator [33]). We found that the posterior could accurately be reconstructed from the final layer of this network, and that the decoding approach was once more the only way that uncertainty could be recovered (S6 Text). However, this network did not qualitatively match the increase in neuronal response gain with BC found in vivo (Fig 4B), possibly hinting that divisive normalization plays a key role in OT activity.

Besides this important computational role for divisive normalization, our model of OT makes two other distinctive predictions. First, the linear decoding of OT responses which we have considered so far should reconstruct the marginalized log-posterior as well as any nonlinear decoder. This is because the small information loss (Fig 3B and 3D) indicates that the neuronal responses are an lPPC, for which all the information available can be extracted linearly. This decoding is furthermore invariant: no additional information about BC is required. Clearly, testing all possible nonlinear decoders is not feasible. Instead, to test this prediction in our model, we trained a deep network (which in principle can approximate any nonlinear decoder) on the OT outputs. We found that the information loss was indistinguishable from that of Fig 3D. A similar approach could be used to assess linearity in experimental recordings of neuronal activity in the OT, but it requires simultaneous recording from a large population of neurons.

**Fig 6:**
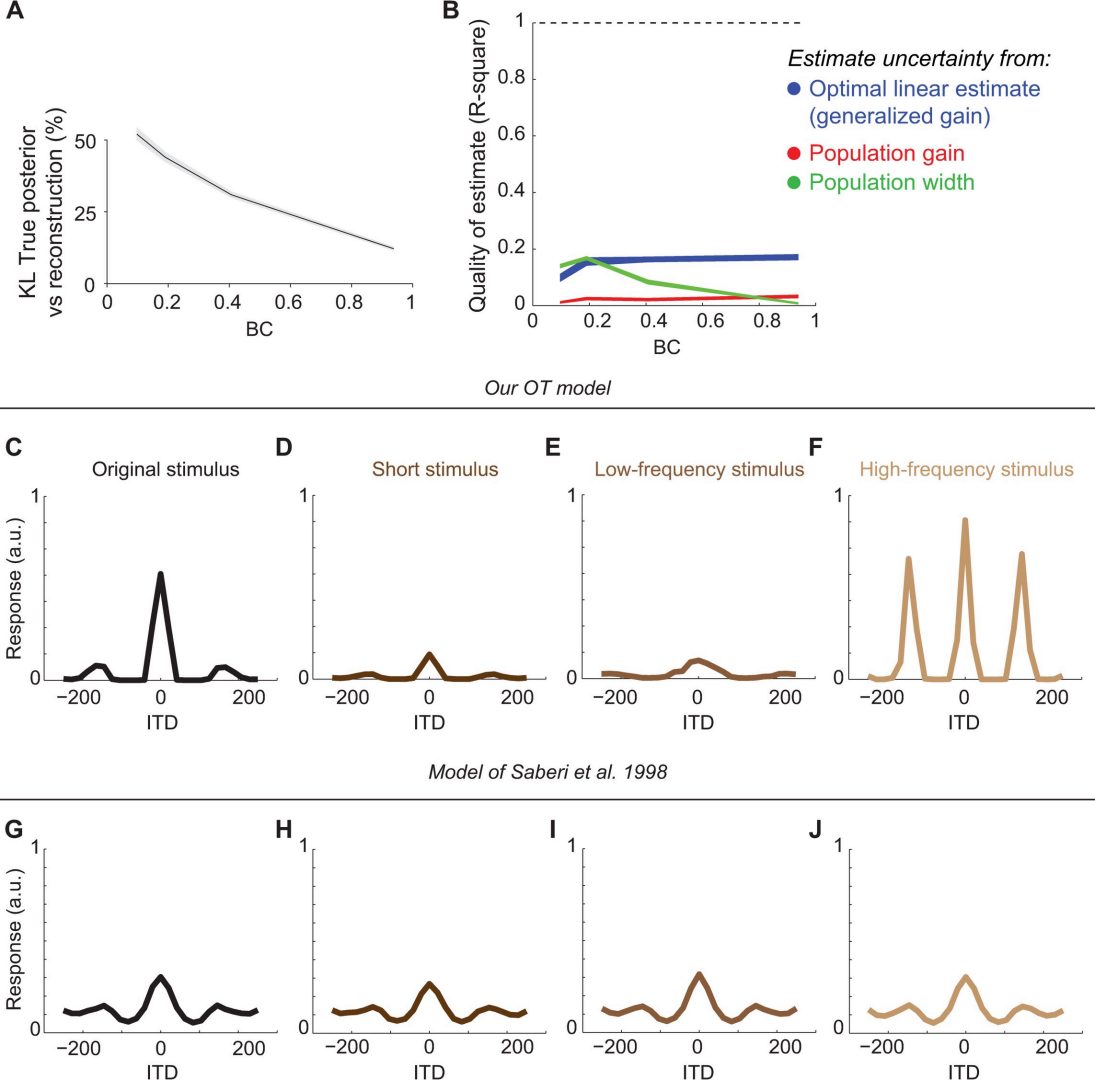
Modulation of OT activity by stimulus reliability. (A,B) Average KL divergence (A) and performance of different estimators of uncertainty (B) from the population activity generated by the model of Saberi et al. [17]. Same conventions as in Fig 3B and Fig 5, respectively. Because this population is not a linear PPC, reconstruction of the uncertainty through linear decoding fails in this example, leading to negative R-square. The corresponding curve was thus removed. (C-F) ITD tuning curves of our OT model for different stimuli. (C) Original stimulus. Tuning curve replotted from Fig 4C-top. (D) Shorter stimulus duration, 5msec vs. 10msec for the original stimulus. (E) Low-passed filtered stimulus. (F) High-pass filtered stimulus. (G-J) Same as (C-F) but for the model of Saberi et al. [17].

A second prediction that could be tested more readily comes from a comparison with descriptive models. For instance, Saberi et al. [17] developed a descriptive model of OT that accounts for the data of Fig 4. In their model, IC is described as computing the cross-correlation between filtered versions of the sound reaching the two ears, and OT activity is given by a linear weighting of IC activity (S7 Text). The model of Saberi et al. differs from ours in two critical ways: first, there is no nonlinear mapping between IC and OT, and second, there is a separate normalization in each frequency band. Both our model and that of Saberi et al. predict qualitatively similar OT tuning curves and dependence of neuronal responses on BC. However, due to the difference between the operations required by the ideal observer and those postulated in the model of Saberi et al., we found that in the latter the linear reconstruction of the log-posterior was poor (Fig 6A), and thus uncertainty could not be estimated accurately (Fig 6B). Is it possible to further distinguish the two models?

Intuitively, neuronal activity in our model should be modulated by any manipulation of the input stimuli that influences the log-posterior, because this is the quantity represented by the neuronal activity. By contrast, the descriptive model of Saberi et al. was designed to fit exclusively neuronal responses to changes in BC, and it is by design sensitive only to the correlations in the different frequency bands in the stimulus. Therefore, we can distinguish the two models by using stimulus manipulations that modify the log-posterior without modifying the correlations within each frequency band. To illustrate this point, we considered stimuli with a shorter duration than those in the experiments of Saberi et al. Shorter stimuli are less informative about ITD. Correspondingly, in our model, OT activity was scaled down compared to the full-length stimuli (Fig 6C and 6D). Second, we changed the frequency content of the stimulus. When we presented a stimulus with only low-frequency components, model responses were scaled down and the side peaks of the tuning curves moved further away from the main peak (a form of tuning widening; Fig 6E), whereas when we presented a stimulus with only high-frequency components we observed the opposite pattern (Fig 6F). As expected, the descriptive model was not sensitive to any of those changes in the stimuli (Fig 6G–6J). This is because the mean correlation inside each frequency band is identical between all the separate conditions. However, the variability across trials of the neuronal responses changed in the different conditions, for instance being larger for short than long stimuli.

In summary, the advantage of the proposed model of OT over descriptive models is that, by postulating a functional goal for OT, it achieves much better generalizability and allows one to make predictions for new experiments. The specific predictions of Fig 6 could be readily tested with current experimental techniques.

## Discussion

We have derived a new ideal observer model of the auditory localization task, and a neural implementation based on lPPCs, that recapitulate several experimental observations on perceptual bias and variability (Fig 2) and on neuronal activity in the auditory pathway (Fig 4). Using this model, we have compared two popular approaches to studying neuronal representations of momentary uncertainty, the Bayesian decoding approach and the correlational approach. Our results highlight a major limitation of the correlational approach. Although specific features of neuronal activity such as tuning width and gain may correlate with stimulus information content and thus with the average uncertainty, they provide poor estimates of momentary uncertainty (Fig 5). This conclusion is robust to model details such as the specific neuronal nonlinearity, which could be optimized to quantitatively fit neuronal or behavioral data.

The Bayesian decoding approach is at the core of a successful theory of probabilistic computation in neuronal populations [8,19,34] and has been applied to estimate the information available in neuronal populations about a stimulus of interest [25–27, 35] and more recently to value-based decision making [36]. Related methods have been proposed recently to identify which directions in population activity space have most influence on behavioral variability [37]. Note that we used the word ‘Bayesian decoding’ in a general sense to not only refer to strictly Bayesian approaches, which invert the true likelihood function through Bayes rules, but also to approximate methods, which invert an approximate likelihood function, as was the case for the lPPC approach. Here we have shown that this Bayesian decoding approach can be used to accurately estimate the sensory uncertainty from the trial-by-trial neural activity. We have also found that the code for uncertainty relies on multiple features of neural activity.

In contrast, the correlational approach partly relies on the intuition that uncertainty should be related to a single feature (or possibly a handful of features) of the neuronal activity, so as to provide a simple code for momentary uncertainty. However, the Bayesian decoding approach argues against this intuition. In order to perform computations that are important for Bayesian inference such as marginalization and multisensory integration, the critical factor is the representation of the posterior distribution, not the uncertainty per se. In that respect, lPPCs, from which the log posterior can be recovered linearly, greatly simplify further inference.

Ultimately, however, a complete comparison of the decoding and correlational approaches has to come from simultaneous measurements of the activity of large neuronal populations and behavioral reports of perceptual uncertainty. Such experiments have yet to be performed. So far, the correlational approach has only been used to demonstrate a correlation between a particular encoding feature (e.g. tuning width) and the experimental variable that controls the average stimulus uncertainty (e.g. BC). The key issue will be to determine whether reports of momentary perceptual uncertainty are related to simple features of neuronal responses, as suggested by the correlational approach, or better predicted by the kind of decoding techniques we have explored here. Note that the correlation approach is not the right way to approach this question, even if it were somehow extended to momentary uncertainty. Indeed, this approach can only identify correlates of momentary uncertainty and might fail to recover how it is decoded in the brain.

Because the data required for such an analysis are not currently available, we have instead followed a normative approach that starts from deriving the ideal observer, namely computing the ground truth posterior distribution for a given sensory stimulus, and using the width of this distribution as an idealized proxy for perceptual uncertainty. This is a standard approach in psychophysics, which has proven helpful by setting an upper bound on what the animal might accomplish [38]. It is possible that the posterior of the ideal observer might not reflect the subjective uncertainty of an animal, and that instead the correlational approach might provide a better approximation of this subjective uncertainty. However, for simple perceptual decision making tasks, it is currently believed that the information available in cortical areas is of the same order as the information available in the behavior, which is to say that the animal’s performance approaches the ideal observer’s performance [12,39]. If future experiments identify a discrepancy between ideal and animal observers, the decoding framework we have illustrated here could provide guidance in identifying the processing stages that are responsible for the discrepancy.

Using the normative approach for ITD processing, we have first shown that a neuronal population of cross-correlation units, commonly used in auditory processing models, provides a close approximation to the true posterior (Fig 3). Note that our model is a variation of the energy models which have been applied to several visual features such as orientation, motion or disparity [40]. It is therefore likely that our conclusions would readily generalize to these other cases. For instance, the standard motion energy model describes the response of a neuron by a pair of spatio-temporal linear filters applied to the visual stimulus, followed by a quadratic nonlinearity applied to each filter’s output, and a linear combination of the results [40]. It has been shown that a suitable linear combination of motion energy units amounts to computing the log-likelihood of the speed of visual motion for a given visual input [41]. This is because the generative model in [41] assumed that the visual input is corrupted by white noise of constant amplitude, thus resulting in a Gaussian likelihood. Similarly, recent work on visual disparity [42] and speed [43] estimation in natural stimuli, used Gaussian likelihoods and thus derived neuronal population representations of the log likelihood using quadratic units (with filters optimized for the specific task). These studies did not address trial-by-trial uncertainty, but given the similarity of the operations involved in the computation of the log likelihood in those models to our pre-marginalization model, a comparison of the decoding and correlational approaches in those models would lead to similar results.

Furthermore, in this work we also went beyond the cross-correlation model, and derived a new and more realistic model of the processing of the ITD cue, which correctly accounts for marginalization of nuisance variables. Besides showing that existing behavioral and neuronal data are well captured by our model (Figs 2 and 4), we have illustrated that by specifying the computational goal for a given neuronal population (in our case, representing the log-posterior in a lPPC), it is possible to generate distinctive predictions about nonlinear response properties of single neurons (Fig 6), which could be readily tested with current recording technologies.

Importantly, our approach also departed from most previous modeling studies of population coding, which start from response statistics that qualitatively match the data, e.g. Bell-shaped tuning curves and limited-range noise correlations [12,44,45]. Such models based on synthetic tuning curves and response variability can lead to unrealistic information content [46], because they may fail to model information-limiting correlations (a.k.a. differential correlations [9]). These correlations are shaped by the statistics of the sensory input [31] and by the computations performed by neural circuits, and are the primary factor influencing information in large neuronal populations. We built instead a circuit that can achieve near optimal performance based on actual sensory input. Such a model may not contain all the correlations found in vivo, such as the ones caused by large common fluctuations [47–50] but, unlike previous models, it is guaranteed to contain information-limiting correlations induced by the input noise (as in [31]). Understanding the neural code for momentary uncertainty requires that these correlations be taken into account.

Our models of IC and OT lack several biological details. For instance, it is known that the topology of IC and OT receptive fields is not uniform: neurons representing the front are more densely packed, and are sensitive to higher frequencies than neurons representing the periphery [51]. This organization of receptive fields might be related to the statistics of natural sounds [52,53]. Here we have ignored this level of detail of the biological system, but an immediate consequence of incorporating such details in our model is that it would lead to a loss of information about ITD, since the neuronal population would be effectively ignoring a range of frequencies that are present in the stimulus. However, the loss of information might be less important when considering sounds with more realistic frequency spectra, as well as the fact that they are filtered by the facial ruff and ear canal before reaching the sensory organ.

Indeed, extending our approach to ITD coding in natural sounds is an important future direction. The statistics of natural sounds support the assumption of independent noise at the two ears [53], thus justifying to some extent the use of white noise stimuli in the experiments we considered. However, sound sources in the natural environment are far from white noise and the auditory localization task involves marginalizing over the unknown frequency spectra of the signal and noise. This problem is computationally intractable, but could be addressed using techniques for approximate inference, such as Expectation Propagation [54,55] or Belief Propagation [56].

## Methods

### Uncertainty in the auditory localization task

We considered the task of localizing a sound source based on the Interaural Time Delay (ITD) *δ* between the sound reaching the left ear and the right ear. More precisely, the task consists of inferring the value of the ITD *δ* given the sounds reaching the two ears: *s*_*R*_,*s*_*L*_. Following classical experiments [17], we considered a stimulus composed of one common white-noise process (the “signal”), plus independent white noise added for each ear (the “noise”). The signal component is offset in the left ear by the ITD (−*σ*):

$$\left\{ \begin{array}{l} s_{R}(t)=\sigma_{S}S(t)+\sigma_{N}\eta_{R}(t)+\sigma_{0}\nu_{R}(t)\\ s_{L}(t)=\sigma_{S}S(t-\delta )+\sigma_{N}\eta_{L}(t)+\sigma_{0}\nu_{L}(t) \end{array}\right.$$ 13

Where *s*(*t*),*η*_*R*_(*t*),*η*_*L*_(*t*),*ν*_*R*_(*t*),*ν*_*L*_(*t*), represent five independent white noise vectors. The parameters *σ*_*S*_ and *σ*_*N*_ control the relative size of the signal and the noise, thus modulating the information content of the stimulus about *δ*. We further added another independent noise with fixed amplitude *σ*_0_ = 0.9, to model internal noise which might be introduced, for example, at the level of hair cells in the cochlea.

Following Saberi et al, 1998, we measure information content using the Binaural Correlation (BC):

$$BC=\frac{\sigma_{S}^{2}}{\sigma_{S}^{2}+\sigma_{N}^{2}}$$ 14

BC = 1 means that the ITD could be estimated perfectly, if not for the internal noise, whereas BC = 0 means there is no information at all about ITD.

We further defined the input, or sensory, uncertainty *v* as the variance of the posterior distribution over *δ* given observation of the sounds reaching the ears *s*_*R*_,*s*_*L*_:

$$v=\mathrm{var}{\left(\delta \right)}_{p\left(\delta |s_{R},s_{L}\right)}^{}$$ 15

Note that this quantity varies on a trial-by-trial basis due to variations in *s*_*R*_,*s*_*L*_.

Below, we show how we computed the posterior distribution first in the case that the BC value is known (pre-marginalization ideal observer), and then in the case that BC is unknown (post-marginalization ideal observer).

For all simulations, the input stimuli were sampled at 48 kHz, and then low-pass filtered with a high frequency cutoff of 8 kHz, representing the fact that owls are receptive only to a finite frequency range. This specific value does not modify our conclusions. For construction of the tuning-curves in Fig 4, input stimuli lasted 9 msec (as in Saberi et al.). For KL and R-square plots, input stimuli lasted instead 1 msec since this decrease of stimulus information led to posterior distributions that were less peaked. We used values *σ*_*N*_ = 0.25,0.77,0.9,0.95 while keeping $\sigma_{S}^{2}+\sigma_{N}^{2}=1$, and varied *δ* from -250 to +250 μsec in steps of 20 μsec. We generated 6,000 stimulus repetitions for each combination of BC and *δ*. Simulations were coded in Matlab.

### Pre-marginalization ideal observer for known BC level

We assume discrete time and use vector notation **s**_*R*_ = (*s*_*R*_(1),*s*_*R*_(2),…,*s*_*R*_(*T*)) and similarly for **s**_*L*_. We further assume that *σ*_*S*_ and *σ*_*N*_ are known, hence we call this ideal observer the pre-marginalization. In practice, an observer would not know in advance the relative levels of the signal and the noise and would have to infer them: we treat this more complex case later.

To perform inference on ITD, the ideal observer needs first to compute the log-likelihood of the inputs as a function of *δ*, and then combine this with the prior in order to compute the posterior. The value for the log-likelihood is:

$$\begin{array}{l} L^{O}\left(\delta ;\sigma_{N},\sigma_{S}\right)=\log p\left(s_{R},s_{L}|\delta ,\sigma_{N},\sigma_{S}\right)\\ \;=-\frac{1}{2}{\left(s_{R},s_{L}\right)}^{⊤}\Sigma_{\delta }^{-1}\left(s_{R},s_{L}\right)-\frac{1}{2}\log\left(\det\left(\Sigma_{\delta }\right)\right)+\mathrm{constant} \end{array}$$ 16

where Σ_*δ*_ is the covariance matrix of the signals (the subscript highlights its dependence on *δ*). If we introduce the symbol **P**_*δ*_ for the permutation matrix that performs the temporal shift by *δ*, namely (**P**_*δ*_**s**_*L*_)(*t*) = *S*_*L*_(*t*−*δ*), then we can write the covariance matrix as follows:

$$\Sigma_{\delta }=\left(\begin{array}{cc} \left(\sigma_{S}^{2}+\sigma_{N}^{2}\right)I_{T} & \sigma_{S}^{2}P_{\delta }^{}I_{T}\\ \sigma_{S}^{2}P_{\delta }^{⊤}I_{T} & \left(\sigma_{S}^{2}+\sigma_{N}^{2}\right)I_{T} \end{array}\right)$$ 17

where $I_{T}$ is the *T*×*T* identity matrix and the off-diagonal terms are the cross-covariances between the two signals. The inverse covariance is then (applying the formula for inverse of block matrices):

$$\Sigma_{\delta }^{-1}=\left(\begin{array}{cc} \frac{\sigma_{S}^{2}+\sigma_{N}^{2}}{\sigma_{N}^{4}+2\sigma_{S}^{2}\sigma_{N}^{2}}I_{T} & -\frac{\sigma_{S}^{2}}{\sigma_{N}^{4}+2\sigma_{S}^{2}\sigma_{N}^{2}}P_{\delta }^{⊤}I_{T}\\ -\frac{\sigma_{S}^{2}}{\sigma_{N}^{4}+2\sigma_{S}^{2}\sigma_{N}^{2}}P_{\delta }^{}I_{T} & \frac{\sigma_{S}^{2}+\sigma_{N}^{2}}{\sigma_{N}^{4}+2\sigma_{S}^{2}\sigma_{N}^{2}}I_{T} \end{array}\right)$$ 18

This leads to the following expression for the log-likelihood:

$$L^{O}\left(\delta ;\sigma_{N},\sigma_{S}\right)=\frac{\sigma_{S}^{2}}{\sigma_{N}^{4}+2\sigma_{S}^{2}\sigma_{N}^{2}}s_{R}^{⊤}P_{\delta }^{⊤}s_{L}+K\left(\sigma_{N},\sigma_{S}\right)$$ 19

where *K* comprises terms that are independent of *δ*. Note that $\left(P_{\delta }^{⊤}s_{L}\right)(t)=S_{L}(t+\delta )$. As expected, the log-likelihood is highest on average when *δ* matches the true ITD. Therefore, for known *σ*_*S*_ and *σ*_*N*_, the ideal observer simply needs to compute the cross-covariance between the input signals (namely, $CC(\delta )=s_{R}^{⊤}P_{\delta }^{⊤}s_{L}$) at all possible relative ITDs, and scale it by $\frac{\sigma_{S}^{2}}{\sigma_{N}^{4}+2\sigma_{S}^{2}\sigma_{N}^{2}}$.

### Ideal observer with marginalization of the BC level

We consider now the more realistic case, in which *BC* is varied across trials and the subject does not have access to the exact *BC* value on each trial. In this case *σ*_*S*_ and *σ*_*N*_ play the role of nuisance variables. To perform inference on ITD, the ideal observer needs to compute the log-likelihood of the inputs as a function of *δ* by marginalizing out *σ*_*S*_ and *σ*_*N*_:

$$L^{M}\left(\delta \right)=\log\left[\int \exp\{L^{O}\left(\delta ;\sigma_{N},\sigma_{S}\right)\}p\left(\sigma_{N},\sigma_{S}\right)d\sigma_{N}d\sigma_{S}\right]$$ 20

Therefore, we first need to expand Eq 20 and write down explicitly the full log-likelihood (up to terms independent of *δ*,*σ*_*S*_,*σ*_*N*_):

$$\begin{array}{l} L^{O}\left(\delta ;\sigma_{N},\sigma_{S}\right)=-\frac{1}{2}{\left(s_{R},s_{L}\right)}^{⊤}\Sigma_{\delta }^{-1}\left(s_{R},s_{L}\right)-\frac{1}{2}\log\left(\det\left(\Sigma_{\delta }\right)\right)\\ \;=-\frac{1}{2}\frac{\sigma_{S}^{2}+\sigma_{N}^{2}}{\sigma_{N}^{4}+2\sigma_{S}^{2}\sigma_{N}^{2}}\left(s_{R}^{⊤}s_{R}+s_{L}^{⊤}s_{L}\right)+\frac{\sigma_{S}^{2}}{\sigma_{N}^{4}+2\sigma_{S}^{2}\sigma_{N}^{2}}\left(s_{R}^{⊤}P_{\delta }^{⊤}s_{L}\right)-\frac{T}{2}\log\left(\sigma_{N}^{4}+2\sigma_{S}^{2}\sigma_{N}^{2}\right) \end{array}$$ 21

We now show that the above is the logarithm of the product of two Gamma distributions. First, we define the following terms:

$$\begin{array}{c} \beta_{1}=\frac{\sigma_{S}^{2}+\sigma_{N}^{2}}{\sigma_{N}^{4}+2\sigma_{S}^{2}\sigma_{N}^{2}}\;;\;\beta_{2}=\frac{\sigma_{S}^{2}}{\sigma_{N}^{4}+2\sigma_{S}^{2}\sigma_{N}^{2}}\\ CC(\delta )=s_{R}^{⊤}P_{\delta }^{⊤}s_{L}\;;\;V=\frac{s_{R}^{⊤}s_{R}+s_{L}^{⊤}s_{L}}{2} \end{array}$$ 22

Finally, by completing the terms in Eq 21, we obtain:

$$\begin{array}{l} L^{O}\left(\delta ;\sigma_{N},\sigma_{S}\right)=-\left(\beta_{1}-\beta_{2}\right)\left(\frac{V+CC(\delta )}{2}\right)-\left(\beta_{1}+\beta_{2}\right)\left(\frac{V-CC(\delta )}{2}\right)\\ \;+\frac{T}{2}\log\left(\beta_{1}-\beta_{2}\right)+\frac{T}{2}\log\left(\beta_{1}+\beta_{2}\right)\\ \;=\log\left[Gam\left(\beta_{1}-\beta_{2};\frac{T}{2},\frac{V+CC(\delta )}{2}\right)Gam\left(\beta_{1}+\beta_{2};\frac{T}{2},\frac{V-CC(\delta )}{2}\right)\right]\\ \;=\log\left[Gam\left(\beta_{1}-\beta_{2};\frac{T}{2},V\frac{1+CC(\delta )/V}{2}\right)Gam\left(\beta_{1}+\beta_{2};\frac{T}{2},V\frac{1-CC(\delta )/V}{2}\right)\right] \end{array}$$ 23

where the second equality holds up to terms independent of *β*_1_,*β*_2_. *Gam* denotes the unnormalized density of a Gamma random variable.

By combining Eq 20 and Eq 23, we thus find that the log-likelihood of *δ*, and thus also the log-posterior, can be expressed as a complex non-linear transformation of the cross-correlation *C*(*δ*)/*V*, up to terms constant in *δ*:

$$\begin{array}{l} L^{M}\left(\delta \right)=\log\left[\int {}_{\beta_{1},\beta_{2}\in [0,\infty [}Gam\left(\beta_{1}-\beta_{2};\frac{T}{2},V\frac{1+CC(\delta )/V}{2}\right)Gam\left(\beta_{1}+\beta_{2};\frac{T}{2},V\frac{1-CC(\delta )/V}{2}\right)\right]\\ \;=\log\left[h\left(\frac{CC(\delta )}{V}\right)\right] \end{array}$$ 24

Now the integral in Eq 20 can be (approximately) solved in closed form if we assume an uniform prior on for *β*_1_−*β*_2_ and *β*_1_+*β*_2_. Let us first compute the integral over all values of *β*_1_, *β*_2_:

$$\log\int_{\beta_{1},\beta_{2}\in ]-\infty ,\infty [}\left[Gam\left(\beta_{1}-\beta_{2};\frac{T}{2},\frac{V+CC(\delta )}{2}\right)Gam\left(\beta_{1}+\beta_{2};\frac{T}{2},\frac{V-CC(\delta )}{2}\right)\right]=-\frac{T}{2}\log\left(1-\frac{CC{\left(\delta \right)}^{2}}{V^{2}}\right)$$ 25

However, this does not completely solve the problem since, by definition, *β*_1_ and *β*_2_ must respect the fact that they are both positive quantities. The integral in Eq 20 thus has a second term corresponding to *p*(*β*_1_≥0∧*β*_2_≥0) when *β*_1_+*β*_2_ and *β*_1_−*β*_2_ both have Gamma random distributions.

The computation of log*p*(*β*_1_≥0∧*β*_2_≥0) cannot be done in closed form. However, we can approximate it. Indeed, noting $\gamma_{+\beta_{1}+\beta_{2}}$ and $\gamma_{-=\beta_{1}-\beta_{2}}$, we have:

$$p(\beta_{1}\geq 0\&\beta_{2}\geq 0)=p(\gamma \_+\geq \gamma \_-)$$ 26

Then for any constant *c*:

$$p(\gamma_{+}\geq \gamma_{-})\geq p(\gamma_{+}\geq c)p(c\geq \gamma_{-})$$ 27

which is an analytical value we can express using the lower and upper incomplete gamma functions (or the ‘gamcdf’ function in Matlab). This approximation ignores the following two terms: *p*(*γ*_+_≥*γ*_−_≥*c*) and *p*(*c*≥*γ*_+_≥*γ*_−_). We can thus upper-bound the error of the approximation with: *p*(*γ*_−_≥*c*)+*p*(*c*≥*γ*_+_).

For the value of *c*, we have used the average of the means of *γ*_+_ and *γ*_−_:

$$2c=\frac{T}{V+CC(\delta )}-\frac{T}{V-CC(\delta )}=\frac{2TC(\delta )}{V^{2}-CC^{2}(\delta )}$$ 28

We find that the log-likelihood function of *δ* after marginalization is approximately (up to constant terms):

$$L^{M}\left(\delta \right)=-\frac{T}{2}\log\left(1-\frac{CC{\left(\delta \right)}^{2}}{V^{2}}\right)+\log p(\gamma_{+}\geq c)+\log p(c\geq \gamma_{-})$$ 29

This approximation is good if both *T* and *C*(*δ*) are big to ensure the precision of the approximation, i.e., the approximation will be correct around the values for which *C*(*δ*) is the highest which corresponds to the maximal values of the posterior.

Any Gamma prior on *β*_1_+*β*_2_ and *β*_1_−*β*_2_ would also be conjugate and would just slightly change the values for *T*,*C*(*δ*) in this formula. The uniform prior we have considered is mis-specified, in that it cannot be normalized. It corresponds to an improper prior on *σ*_*S*_, *σ*_*N*_ with density:

$$p(\sigma_{S},\sigma_{N})=\frac{\sigma_{S}}{\sigma_{N}^{3}(\sigma_{N}^{2}+2\sigma_{S}^{2})}$$ 30

However, note that the specific form of the prior we use does not influence excessively Eq 29. This is because the stimulus is quite long and thus quite informative about BC. Prior information thus has low influence.

### Ideal observer behavior

In order to compare the ideal observer models to the behavioral data of Saberi et al [17], we used the following model of the ideal observer behavior. Since the posterior distribution tends to be multimodal, the mean of the posterior tends not to be very representative of the posterior. Instead, we used the maximum a posterior (MAP) estimator. Given a posterior *p*(*δ*), the MAP is given by:

$$\delta_{MAP}=argmax(p(\delta |s_{R},s_{L}))$$ 31

This gives a point estimate of the ITD. In order to transform this value into a head-turn angle, we used the relationship given by Saberi et al to approximate the transformation between ITD and angle *θ*:

δ=Asin(ωθ) 32

With *A* = 260*μs* and *ω* = 0.0143(°)^−1^.

### Neural implementations of the ideal observers

We aimed to build models of neuronal populations that closely approximate the ideal observers, while also incorporating known aspects of auditory physiology.

First, to mimic early auditory processing, we convolved the input signals through a bank of bandpass filters, with four logarithmically spaced center frequencies ranging from 0.5 to 12 kHz. These four filters were built using formulas from Simoncelli et al, 1992 [57] so that they were self-invertible.

A self-invertible family is a family of filters / vectors **F**_*n*_ such that the sum of their squared Fourier transforms sums up to 1 at all frequencies (beneath some cut-off frequency). Invertible families have two useful properties: first, for any input ***s*** with no frequency content above the cut-off of the highest filter (12 kHz), we can decompose this input by filtering it (convolving it) with every member of the family, and reconstruct the original signal by convolving a second time:

$$s_{}=\sum_{n=1}^{4}F_{n}^{⊤}*\left(F_{n}^{⊤}*s_{}\right).$$ 33

Second, we can also construct the covariance between two signals by summing the covariance of the filtered signals:

$$\sum_{t=0}^{T}s_{R}(t)s_{L}(t)=\sum_{t=0}^{T}\sum_{n=1}^{4}\left(F_{n}^{⊤}*s_{L}\right)(t)\left(F_{n}^{⊤}*s_{R}\right)(t)$$ 34

These two properties are specific to invertible families. For other families of filters, correction terms would have to be added.

Armed with such filters, we then introduced a range of possible delays (between -250 and 250) in the left ear signals, and computed the cross-covariance between the filtered, shifted signals at the two ears, within each frequency band and at each relative delay. Finally, we rectified these signals to produce positive firing rates, thus modeling neuronal activity in the IC. Note that the output of the convolution of the inputs with each filter is a vector with as many dimensions as there are samples in the input signal (480, see Section “Uncertainty in the auditory localization task”). Therefore, we had a population of 21,120 neurons (4 frequencies × 11 delays × 480 time samples). To reduce the memory requirements, we summed the responses across time points, and verified that this did not change substantially any of the results. In conclusion, the response of an IC neuron with preferred delay *δ*_*n*_ and filter **F**_*n*_ was:

$$r_{n}^{\mathrm{IC}}\left(s_{R},s_{L}\right)=\sum_{t=0}^{T}{\left\lfloor \left(F_{n}^{⊤}*P_{\delta_{n}}^{⊤}s_{L}\right)\left(F_{n}^{⊤}*s_{R}\right)\right\rfloor }_{+}(t)$$ 35

Note that this model does not include Poisson variability, such as that due to the spiking mechanism or stochastic network dynamics. Such variability would not affect our results, as it can always be averaged away in neuronal populations that are large enough [50].

We then considered what additional operations should be performed by downstream populations to implement the ideal observers. Specifically, we required that the log-posterior, $\log\left[p\left(\delta |s_{R},s_{L}\right)\right]$, can be linearly reconstructed from the neuronal population activity:

$$\log\left[p\left(\delta |s_{R},s_{L}\right)\right]=\sum_{n}h_{n}\left(\delta \right)r_{n}\left(s_{R},s_{L}\right)+\mathrm{const}$$ 36

The terms *h*_*n*_(*δ*) are the (stimulus-dependent, i.e. *δ*-dependent) reconstruction weights, and it is the task of a downstream area to learn such weights in order to correctly read out the population activity (see Section “Decoding uncertainty from population activity”). When Eq 36 is satisfied exactly, the population is said to implement the ideal observer in a linear Probabilistic Population Code (lPPC).

For the pre-marginalization ideal observer, Eq 19 implies that such a lPPC is obtained by computing the frequency-independent cross-covariance between the input signals at all possible ITDs. Because we used invertible filters, our IC population model, prior to rectification, would implement a lPPC simply by summing across frequencies with the correct weights (see Eq 33). However, due to the rectification, the IC population is not exactly a lPPC, and we quantified its deviation as described in Section “Decoding uncertainty from population activity”.

For the ideal observer with unknown BC, Eq 29 shows that additional computation needs to be performed on the outputs of the IC model, to obtain a lPPC of the ideal observer. This computation involves the steps illustrated in Fig 3C: summing the cross-covariance terms across frequencies, divisive normalization to obtain the cross-correlation, and the nonlinearity of Eq 29. This would lead to a so-called delta-kernel lPPC, where each neuron enters Eq 36 with weight 1 for its preferred *δ* and 0 otherwise. We considered an additional filtering stage, using invertible, shift-invariant filters to obtain smooth tuning curves to ITD. For mathematical convenience, we did this using a pair of self-invertible filters *F*_1_, *F*_2_. The neuronal activity given the pre-marginalization log-posterior log*p*(*δ*|**s**_*R*_,**s**_*L*_) is given by convolving this function with the self-invertible pair. Our model is thus separated between two sub-populations with slightly different tuning-properties. Note that this separation in two sub-populations is done purely for mathematical convenience and should not be interpreted as a prediction for neuronal activity in vivo. Indeed, there are many alternate possibilities for representing the log-posterior in neuronal activity that might give quantitatively more accurate accounts of electrophysiological data. Namely, the filters that transform the log-likelihood into neuronal activity could be optimized to fit the activity of neurons recorded experimentally.

Finally, we added a rectification step to enforce positive firing rates. To summarize, the response of an OT neuron with preferred delay *δ*_*n*_ was modeled as:

$$r_{n}^{OT}=\left\lfloor \sum_{\delta }L^{M}\;\left(\delta \right)H_{n}(\delta )\right\rfloor +(t)$$ 37

where *H*_*n*_ is the filter for neuron *n*. By construction, this model, prior to rectification, would implement the marginalized ideal observer in a lPPC: $L^{M}$ can be computed simply by inverting the filter matrix *H* (which, since the filters are once again self-invertible, is *H* itself). We quantified the deviation from the ideal observer due to the rectification as explained in Section “Decoding uncertainty from population activity”.

### Decoding uncertainty from population activity

We defined the decoding approach as the problem of reconstructing the posterior distribution over *δ* from the activity of a neuronal population. In this approach, there is then a natural definition of the uncertainty as the posterior variance. We then asked whether it is possible to estimate the uncertainty on a trial-by-trial basis from neuronal recordings.

As explained above, we assumed that the log-posterior could be obtained through weighted sums of neuronal responses (Eq 36). To estimate the decoding weights, we trained a multinomial logistic regression model (using the ‘maxent’ Matlab library [58]) with predictors $\left\{r_{n}^{IC}\right\}$ or $\left\{r_{n}^{OT}\right\}$, and discrete classes labeled by discretized values of *δ* (25 values spanning the [−250*μs*, 250*μs*] range; 21*μs* between successive values). The most common usage of multiclass logistic regression is to provide only the class labels (not the full distribution over classes) for the training examples, and define as objective function the cross-entropy between the Dirac delta distribution at the true class label and the predicted distribution over classes. This is also equal to the Kullback-Leibler (KL) divergence between those distributions. Here instead we define as objective function the KL divergence between the true posterior (Eqs 19 and 29, for the pre- and post-marginalization cases, respectively) and the reconstructed posterior. This is sometimes referred to as training with probabilistic feedback [59]. Note that, if a neuronal population is a lPPC, then multinomial logistic regression gives the optimal decoder if supplied with a sufficient amount of training examples. Here we optimized the weights on a training set comprising 3,000 trials for each value of *δ* and BC, and then evaluated the reconstruction quality on an independent, equally sized test set. Note also that for the IC model population, a different set of weights was learned for each value of BC. For the OT population, a single set of weights was learned across all values of BC.

For the evaluation, we computed the KL divergence between the true posterior *p*(*δ*|*s*_*R*_,*s*_*L*_) computed by the ideal observer, and the reconstruction. A value of KL close to zero means that the neural responses are close to a lPPC implementation of the ideal observer. We further normalized the KL by dividing it by the KL between the true posterior and the prior, and expressed the result as a percent information loss [50].

In addition, in order to provide a direct comparison between the decoding and correlational approaches, we investigated whether the variance of the reconstructed posterior could be used to estimate the ideal observer’s uncertainty. We quantified estimate quality by the R^2^ on the test set.

## Supporting information

S1 Text Impact of a Gaussian prior. (DOCX)

S2 Text Difference in behavior between pre- and post-marginalization ideal observer. (DOCX)

S3 Text Ideal observer behavior is biased because of edge-effects. (DOCX)

S4 Text Comparison of different non-linearities in the IC model. (DOCX)

S5 Text Other predictors and measures of uncertainty. (DOCX)

S6 Text Approximating the marginalization with a cascade of rectified-linear neurons. (DOCX)

S7 Text Alternative descriptive models of IC and OT. (DOCX)

S8 Text Variance is a natural measure of uncertainty. (DOCX)

S9 Text Supplementary information bibliography. (DOCX)

S1 Fig Difference in behavior caused by multiple manipulations. (A,B) mean and standard deviation of the Maximum A Posteriori (MAP) estimate of the angle of the sound source for the post-marginalization ideal observer with a box prior (reproduced from main text Fig 2). Four different values of the true angle are shown (dashed lines). At low BC, the behavior is strongly biased towards 0, the center of the prior belief of the observer, whereas at high BC it correctly recovers the correct value. (C,D) behavior of the post-marginalization ideal observer when the box prior is replaced with the Gaussian prior of Fischer and Pena, 2011. The mean behavior is almost identical, while the standard deviation of the behavior is reduced at low values of BC (E,F) behavior of the pre-marginalization ideal observer with the Gaussian prior of Fischer and Pena 2011. At low values of BC, the pre-marginalization ideal observer mostly ignores the data, resulting in stronger bias of the mean behavior and lower standard deviation at low values of BC. At high values of BC, the post-marginalization ideal observer correctly estimates BC and both observers have very similar behavior.
(TIF)

S2 Fig Effect of prior range on behavioral bias. (A,B) The mean behavior of the Bayesian ideal observer (post-marginalization) is biased at lower values of BC. This bias is towards the center of the prior: in the realistic case, 0, (box prior over [−250*μs*, 250*μs*] range; panel A), and towards 100 for an unbalanced prior (box prior over [−250*μs*, 500*μs*]; panel B).
(TIF)

S3 Fig Comparison of different nonlinearities in the IC model. Each column correspond to a different model, with the static nonlinearity indicated at the top. (A) Width (top row) and inverse gain (bottom) of the tuning curves, as a function of BC, expressed as a percent change from the value measured at the highest BC. Gray symbols: models. Blue: data from Cazettes et al.. (B) Average KL divergence between the posterior distribution computed by the ideal observer and the posterior decoded from the model population activity. Same conventions as in Fig 3B. (C) Performance of different estimators of uncertainty. Same conventions as in Fig 5A. Notice how, even in the sigmoid model in which there are large changes of width across different values of BC, the width gives poor reconstructions of the uncertainty.
(TIF)

S4 Fig Additional comparisons of the decoding and correlational approaches with additional neuronal activity features. A. Comparison in the auditory model (as in Fig 3D), with two additional features: all products of neuronal activity (as a surrogate of neuronal correlations), and the gain/width ratio of the neuronal activity. Uncertainty is defined as the log-variance as in the main text. The optimal bilinear estimate overfits at the largest value of BC thus leading to a negative R-square value. B. Same as A but uncertainty is defined as the posterior log-entropy instead.
(TIF)

S5 Fig A trained feedforward network performs BC marginalization. (A) Schematic of the deep network architecture. The marginal posterior is approximated from the IC model activity by four layers of neurons implementing a rectifying nonlinearity. The weight matrices W are trained to achieve the best approximation. (B) Average KL divergence between the posterior distribution computed by the ideal observer and the posterior decoded from the model population activity. Same conventions as in Fig 3B. (C) Performance of different estimators of uncertainty. Same conventions as in Fig 5.
(TIF)
