# Supplementary material for: Investigating the representation of uncertainty in neuronal circuits
Source: PLoS Comput Biol. 2021 Feb 12;17(2):e1008138. doi: 10.1371/journal.pcbi.1008138 (PMC7880493; doi:10.1371/journal.pcbi.1008138)
Supplement: S1 Text — (DOCX) [file pcbi.1008138.s001.docx]

## 1. Impact of a Gaussian prior

Fischer and Pena, 2011, proposed to model owl-behavior using a Gaussian prior instead of a box prior like ours. We thus checked how much impact the prior has on our model of the behavior.

As can be seen in supplementary figure 1, the behavior is, as expected, only different for low values of BC. Indeed, at high levels of BC, the information provided by the stimulus overwhelms the information provided by the prior and the prior thus stops to have an influence on the behavior. At low values of BC, the presence of a Gaussian prior increases the bias of the animal towards central angles. This is most apparent in the standard deviation of the behavior which is smaller in the presence of a Gaussian prior.
