# Supplementary material for: Investigating the representation of uncertainty in neuronal circuits
Source: PLoS Comput Biol. 2021 Feb 12;17(2):e1008138. doi: 10.1371/journal.pcbi.1008138 (PMC7880493; doi:10.1371/journal.pcbi.1008138)
Supplement: S2 Text — (DOCX) [file pcbi.1008138.s002.docx]

## 2. Difference in behavior between pre and post-marginalization ideal observers

In the main text, we have shown that the post-marginalization ideal observer fits owl behavior in the experiments of Saberi et al. (Ref. [16] in the main text). The pre and post-marginalization ideal observers have slightly different behaviors, particularly for low BC, as shown in Supplementary Fig. 1 below. This difference in behavior is due to the fact that the pre-marginalization observer knows the exact value of BC on each trial. Therefore, when BC is low and the stimulus not informative, the pre-marginalization ideal observer relies almost entirely on prior information to guess the true value of the angle of the incoming sound. In contrast, the post-marginalization observer puts more weight on the information provided by the sound. This makes the post-marginalization behavior noisier and more quickly biased away from the prior information. Critically, this difference in behavior is only visible in the presence of a more informative prior than the box prior which we assumed which then dominates the low information supplied by the stimulus. Supplementary fig. 1 panels (E,F) thus correspond to the behavior of the pre-marginalization ideal observer with the Gaussian prior of Fischer and Pena, 2011.
