# Supplementary material for: Investigating the representation of uncertainty in neuronal circuits
Source: PLoS Comput Biol. 2021 Feb 12;17(2):e1008138. doi: 10.1371/journal.pcbi.1008138 (PMC7880493; doi:10.1371/journal.pcbi.1008138)
Supplement: S3 Text — (DOCX) [file pcbi.1008138.s003.docx]

## 3. Ideal observer behavior is biased because of edge-effects

In our simulations, the behavior of the Bayesian ideal-observer post-marginalization matched the behavioral data from Saberi et al. (Ref. [16] in the main text). In particular, it matched the bias towards $\theta=0$ (corresponding to a sound source straight ahead) when the value of BC was small. This bias was caused by the edge-effects introduced by our box prior over possible ITD values which spanned the range $\left[ -250\mu s,250\mu s \right]$. Indeed, consider the case in which the true value corresponds to $200\mu s$. Because of the prior, overshooting by more than 50ms is impossible, while undershooting by as much as $400\mu s$ is possible. This distorts the mean behavior of the ideal observer towards the center of the box prior when the estimation is very noisy, i.e. at low values of BC for which the information-content of the stimulus is low.

We confirmed this explanation by performing simulations with an unrealistic prior which was not centered around 0. This unbalanced prior spanned the range $\left[ -250\mu s,500\mu s \right]$, centered instead at $125\mu s$. Under this new prior, we found that the bias towards $\delta=0$ was indeed shifted to instead be around 125 as shown in Supplementary Fig. 2 below.

Note that panel A differs from the corresponding panel in main text Fig. 2. This is because Supplementary Fig. 2 presents the ITD $\delta$ rather than the angle $\theta$. We used the ITD because the new range of possible ITD values under the unbalanced prior does not necessarily correspond to a possible angle.
