# Supplementary material for: Investigating the representation of uncertainty in neuronal circuits
Source: PLoS Comput Biol. 2021 Feb 12;17(2):e1008138. doi: 10.1371/journal.pcbi.1008138 (PMC7880493; doi:10.1371/journal.pcbi.1008138)
Supplement: S4 Text — (DOCX) [file pcbi.1008138.s004.docx]

## 4. Comparison of different non-linearities in the IC model

One difference between our model and that of Cazettes et al. 2016 is the static non-linearity: half-rectification in our case, versus exponential in Cazettes et al. which provides a good fit to intracellular recordings in the external nucleus of IC (ICx).

We therefore reproduced our analysis with two additional non-linearities: a sigmoid nonlinearity similar to Fischer et al., and an exponential nonlinearity similar to Cazettes et al. First, we analyzed how neuronal tuning was affected by changes in stimulus information content (Fig 6A). In all models, gain decreased linearly with BC, whereas width decreased faster and saturated around BC=0.4, in qualitative agreement with the physiology. Furthermore, we found that the model with sigmoid nonlinearity matched the physiology better, exhibiting a stronger modulation of both gain and width across BC.

However, these differences in neuronal responses did not have a substantial effect on the quality of the reconstruction of the pre-marginalization ideal observer from IC activity (Fig 6B). Moreover, while the estimates of the uncertainty of the pre-marginalization ideal observer from width were better with the sigmoid nonlinearity than with half rectification (compare the green lines in the right and left plots in Fig 6C), they remained well below those obtained from the reconstructed posterior (black line Fig 6C). Therefore, the choice of the specific nonlinearity is unlikely to be the reason of the difference between our results and those of Cazettes et al. Rather, our analysis showed that even when width correlated with BC, and therefore with the average uncertainty across trials, it did not correlate well with uncertainty on a trial-by-trial basis.

These results suggest that the detailed changes in tuning curve shape are not the key feature of the representation of uncertainty in any of those models. Population responses contain information about uncertainty than can be extracted by decoding, but is easily lost when focusing only on specific response features.
