# Supplementary material for: Investigating the representation of uncertainty in neuronal circuits
Source: PLoS Comput Biol. 2021 Feb 12;17(2):e1008138. doi: 10.1371/journal.pcbi.1008138 (PMC7880493; doi:10.1371/journal.pcbi.1008138)
Supplement: S5 Text — (DOCX) [file pcbi.1008138.s005.docx]

## 5. Other predictors and measures of uncertainty

In our work, we mostly focused on the variance as a measure of the spread of the posterior distribution of the ideal observer. While this is a choice that is coherent with the literature, it is also not the only possibility. We thus checked whether other measures of uncertainty would change our conclusions. In particular, we could use the entropy of the posterior distribution as a measure of how spread it is. This measure would be less sensitive than the variance to whether the posterior is unimodal or multimodal. We thus repeated our comparison of the decoding and correlational approaches but this time targeting the entropy of the posterior as the uncertainty to be estimated (Fig. S4B). We also checked whether the ratio of the population gain divided by the population width would give a better predictor to the other predictors we had tested so far.

Furthermore, Fisher information and neuronal correlations have also been linked to uncertainty in theoretical studies. Unfortunately, Fisher information isn’t defined on a trial-by-trial basis. It is thus impossible to try to predict uncertainty from the Fisher information present in a given trial. In contrast, it is possible to use the empirical correlation as a surrogate for the true correlation. We thus tried whether the reconstruction would be improved by the inclusion of the products of the activities of neurons $i$ and $j$ on top of the activity of each neuron (pink lines in Fig. S4).

The results are reported in Supplementary Figure 4. We found that, while the quantitative picture is slightly changed, the qualitative conclusions still hold: the decoding approach is the best approach for estimating the uncertainty, no matter the particular measure of uncertainty or feature we use.
