# Supplementary material for: Investigating the representation of uncertainty in neuronal circuits
Source: PLoS Comput Biol. 2021 Feb 12;17(2):e1008138. doi: 10.1371/journal.pcbi.1008138 (PMC7880493; doi:10.1371/journal.pcbi.1008138)
Supplement: S6 Text — (DOCX) [file pcbi.1008138.s006.docx]

## 6. Approximating the marginalization with a cascade of rectified-linear neurons.

Our model of OT requires a very specific non-linearity between input and output. In our model, we just assumed that this non-linearity could be implemented in a single neuron. In order to check whether our results were robust, we furthermore checked that they would hold if the required non-linearity was implemented by multiple layers of neurons with a simpler non-linear input-output relationship.

More specifically, we trained a feed-forward neuronal population, in which neurons were organized hierarchically and used a rectifying nonlinearity, to compute the marginal log-posterior (see next section for details). In order to train the model network, we generated IC activity as described in the main text. We then trained a deep-network to reconstruct the marginal log-likelihood. The neurons computed a simple linear-rectification of their inputs. The architecture of the network was as follows: the input layer was comprised of the cross-covariance across all possible preferred lags and frequencies and of the variance across all frequencies. We then stacked four fully connected layers of 50 neurons on top of this input layer. The final layer provided a multiclass logistic regression of the second-to-last layer. The objective function of the deep-network was to minimize the KL divergence between the true marginal posterior and its multiclass logistic decoding in the last layer. This network could thus be said to implement a lPPC in its second-to-last layer. The training consisted of stochastic gradient descent over batches of training examples. Performance was assessed on a testing data set in order to avoid over fitting, though this hardly mattered.

Once the training was completed, we compared, in a separate validation dataset, the true posterior to its approximation by the deep-network. We measured the percentage KL-loss in order to assess the relative performance of the deep-net compared to observing the true posterior. We also measured the quality of the varying reconstructions of the true uncertainty using the R^2^ value.

We found that a population of only 225 neurons, organized in four hierarchical layers, achieved almost perfect estimation of the posterior (Supplementary Fig. 5A,B). This is not very surprising given that feed-forward networks of more than two layers can approximate any function [S1, S2].

We then asked whether we could estimate uncertainty accurately from this neuronal population. Supplementary Figure 5C shows that, similar to the other models considered so far, the uncertainty of the post-marginalization ideal observer could be more accurately estimated from approximate decoding than from any specific features of neuronal activity. We noted however that the reconstruction was not perfect at high BC. This was due to some individual cases in which the reconstruction is very close to the true one, but has occasional small secondary peaks. As discussed above. for high BC, where the true posterior has variance close to zero, this leads to an overestimation of the variance in the reconstruction. This effect was more pronounced for the trained network than for our OT model, leading to lower R-square values in Supplementary Fig. 4C than in main text Fig. 5B. Multimodal posteriors are indeed one case in which posterior variance may not be the best measure of uncertainty [S2, S3]. However, for downstream computation, like marginalization, what matters is the accuracy of the reconstruction of the posterior, which is better measured by the normalized KL.

These results indicate that while the ideal observer solution for building a lPPC of sound location in the OT can be constructed with divisive normalization (Fig. 3), a nonlinearity which is commonly found in neural circuit [S4] and has been related to marginalization [S5], other implementations are possible such as one relying on a cascade of simpler nonlinearities. However, we found that this gives a poorer account of OT activity than our direct model: the trained network did not predict the increase in gain with BC found in vivo (main text Fig. 4B), possibly hinting that divisive normalization plays a key role in OT activity.

Supplementary Figure 5. A trained feedforward network performs BC marginalization. (A) Schematic of the deep network architecture. The marginal posterior is approximated from the IC model activity by four layers of neurons implementing a rectifying nonlinearity. The weight matrices W are trained to achieve the best approximation. (B) Average KL divergence between the posterior distribution computed by the ideal observer and the posterior decoded from the model population activity. Same conventions as in main text Figure 3B. (C) Performance of different estimators of uncertainty. Same conventions as in main text Figure 5.

S1. Hornik, K., Stinchcombe, M. & White, H. Multilayer feedforward networks are universal approximators. *Neural networks* **2**, 359-366 (1989).

S2. Orhan, A. E. & Ma, W. J. Efficient probabilistic inference in generic neural networks trained with non-probabilistic feedback. *Nature Communications* **8** (2017).

S3. Pouget, A., Drugowitsch, J. & Kepecs, A. Confidence and certainty: distinct probabilistic quantities for different goals. *Nat Neurosci* **19**, 366-374, doi:10.1038/nn.4240 (2016).

S4. Carandini, M. & Heeger, D. J. Normalization as a canonical neural computation. *Nature Reviews Neuroscience* **13**, 51-62 (2012).

S5. Beck, J. M., Latham, P. E. & Pouget, A. Marginalization in neural circuits with divisive normalization. *The Journal of neuroscience : the official journal of the Society for Neuroscience* **31**, 15310-15319, doi:10.1523/JNEUROSCI.1706-11.2011 (2011).
