# Supplementary material for: Investigating the representation of uncertainty in neuronal circuits
Source: PLoS Comput Biol. 2021 Feb 12;17(2):e1008138. doi: 10.1371/journal.pcbi.1008138 (PMC7880493; doi:10.1371/journal.pcbi.1008138)
Supplement: S7 Text — (DOCX) [file pcbi.1008138.s007.docx]

## 7. Alternative descriptive models of IC and OT

We considered also two descriptive models of IC and one of OT that have been proposed in the past based on the physiology.

First, we implemented a simplified version of the model of Fischer et al., 2009, with input gain control, divisive normalization and a sigmoid nonlinearity:

$$\begin{matrix} r_{n}^{IC2009}=\sum_{t=0}^{T} \sigma\left(\frac{u_{n}^{L}(t)u_{n}^{R}(t)}{\frac{1}{2}\sqrt{\left(u_{n}^{L}(t)\right)^{2}+\left(u_{n}^{R}(t)\right)^{2}}}\right) \\ \sigma(x)=\frac{a}{1+\exp\left(-\alpha x+\beta\right)} \\ u_{n}^{L}(t)=\frac{\left(\mathbf{F}_{n}^{\top}*\mathbf{P}_{\delta_{n}}^{\top}\mathbf{s}_{L}\right)(t)}{\sqrt{1+\left(\mathbf{F}_{n}^{\top}*\mathbf{P}_{\delta_{n}}^{\top}\mathbf{s}_{L}\right)^{2}(t)}} \\ u_{n}^{R}(t)= \frac{\left(\mathbf{F}_{n}^{\top}*\mathbf{s}_{R}\right)(t)}{\sqrt{1+\left(\mathbf{F}_{n}^{\top}*\mathbf{s}_{R}\right)^{2}(t)}} \end{matrix}$$

Second, we implemented a model similar to Cazettes et al., 2016, with divisive normalization and exponential nonlinearity:

$r_{n}^{IC2016}=\sum_{t=0}^{T} \exp\left(\alpha\frac{\left(\mathbf{F}_{n}^{\top}*\mathbf{P}_{\delta_{n}}^{\top}\mathbf{s}_{L}\right)\left(\mathbf{F}_{n}^{\top}*\mathbf{s}_{R}\right)(t)}{\frac{1}{2}\sqrt{\left[\left(\mathbf{F}_{n}^{\top}*\mathbf{P}_{\delta_{n}}^{\top}\mathbf{s}_{L}\right)(t)\right]^{2}+\left[\left(\mathbf{F}_{n}^{\top}*\mathbf{s}_{R}\right)(t)\right]^{2}}}\right)$

Third, we implemented the OT model of Saberi et al., 1998. We first split the incoming signal using a bank of 15 self-invertible filters. For each output, the signal was then cubically rectified before computing the cross-correlation in each frequency band. The cross-correlation in each frequency band was then collapsed into a single value using the frequency-specific weights described in Saberi et al, 1998. Like in all of our models, we summed the instantaneous activity over time to avoid running out of memory.

$$r_{n}^{saberi}=\sum_{i=1}^{15} W_{i}Corr((\left\lfloor\mathbf{F}_{i}*\mathbf{s}_{R}\right\rfloor_{+})^{3},(\left\lfloor\mathbf{F}_{i}*\mathbf{s}_{L}\right\rfloor_{+})^{3}))$$

Where we have noted $F_{i}$ the $i^{th}$ frequency filter and $W_{i}$ the weights for the frequency convergence.
