# Supplementary material for: Investigating the representation of uncertainty in neuronal circuits
Source: PLoS Comput Biol. 2021 Feb 12;17(2):e1008138. doi: 10.1371/journal.pcbi.1008138 (PMC7880493; doi:10.1371/journal.pcbi.1008138)
Supplement: S8 Text — (DOCX) [file pcbi.1008138.s008.docx]

## 8. Variance is a natural measure of uncertainty

The variance is a natural measure of the uncertainty associated to an estimator (whether Bayesian or not). Indeed, it corresponds to the inverse of the optimal weight one should put on this estimator when combining it with other estimators as we prove in this section.

Consider the following situation. We want to estimate a quantity of interest $\theta$. We have access to two estimators $\hat{\theta}_{1}$ and $\hat{\theta}_{2}$ with respective variances $v_{1}$ and $v_{2}$. What is the best way to combine the information provided by both into a single estimator?

A natural idea consists in taking a weighted average of the two:

$$\hat{\theta}=\alpha\hat{\theta}_{1}+\left( 1-\alpha\right)\hat{\theta}_{2}$$

The variance of this estimator is:

$$\alpha^{2}v_{1}+\left( 1-\alpha\right)^{2}v_{2}$$

The value of $\alpha$ which minimizes this variance is: $\alpha^{*}=\frac{\left( v_{1} \right)^{-1}}{\left( v_{1} \right)^{-1}+\left( v_{2} \right)^{-1}}$. In other words, the estimators need to be weighted by their inverse-variance in order to get the minimum-variance combined estimator.

Note that, if we further know the likelihood of both estimators as a function of the true parameter value $\theta$, then this linear combination is sub-optimal. Deriving an estimator from the joint-likelihood would result in a non-linear estimator with smaller variance. The only exception is if both estimators are Gaussian (Gauss-Markov theorem). Thus, while the variance represents a natural notion of the uncertainty associated with an estimator, this still destroys information that is present in the likelihood.
