# Supplementary material for: Investigating the representation of uncertainty in neuronal circuits
Source: PLoS Comput Biol. 2021 Feb 12;17(2):e1008138. doi: 10.1371/journal.pcbi.1008138 (PMC7880493; doi:10.1371/journal.pcbi.1008138)
Supplement: S9 Text — (DOCX) [file pcbi.1008138.s009.docx]

## 9. Supplementary Information bibliography

1 Cazettes, F., Fischer, B. J. & Pena, J. L. Cue Reliability Represented in the Shape of Tuning Curves in the Owl's Sound Localization System. *The Journal of neuroscience : the official journal of the Society for Neuroscience* **36**, 2101-2110, doi:10.1523/JNEUROSCI.3753-15.2016 (2016).

2 Hornik, K., Stinchcombe, M. & White, H. Multilayer feedforward networks are universal approximators. *Neural networks* **2**, 359-366 (1989).

3 Orhan, A. E. & Ma, W. J. Efficient probabilistic inference in generic neural networks trained with non-probabilistic feedback. *Nature Communications* **8** (2017).

4 Pouget, A., Drugowitsch, J. & Kepecs, A. Confidence and certainty: distinct probabilistic quantities for different goals. *Nat Neurosci* **19**, 366-374, doi:10.1038/nn.4240 (2016).

5 Carandini, M. & Heeger, D. J. Normalization as a canonical neural computation. *Nature Reviews Neuroscience* **13**, 51-62 (2012).

6 Beck, J. M., Latham, P. E. & Pouget, A. Marginalization in neural circuits with divisive normalization. *The Journal of neuroscience : the official journal of the Society for Neuroscience* **31**, 15310-15319, doi:10.1523/JNEUROSCI.1706-11.2011 (2011).

7 Fischer, B. J. & Peña, J. L. Bilateral matching of frequency tuning in neural cross-correlators of the owl. *Biological cybernetics* **100**, 521-531 (2009).

8 Saberi, K. *et al.* Effects of interaural decorrelation on neural and behavioral detection of spatial cues. *Neuron* **21**, 789-798, doi:{10.1016/S0896-6273(00)80595-4} (1998).
