# Supplementary figures and images for: Investigating the representation of uncertainty in neuronal circuits
Source: PLoS Comput Biol. 2021 Feb 12;17(2):e1008138. doi: 10.1371/journal.pcbi.1008138 (PMC7880493; doi:10.1371/journal.pcbi.1008138)

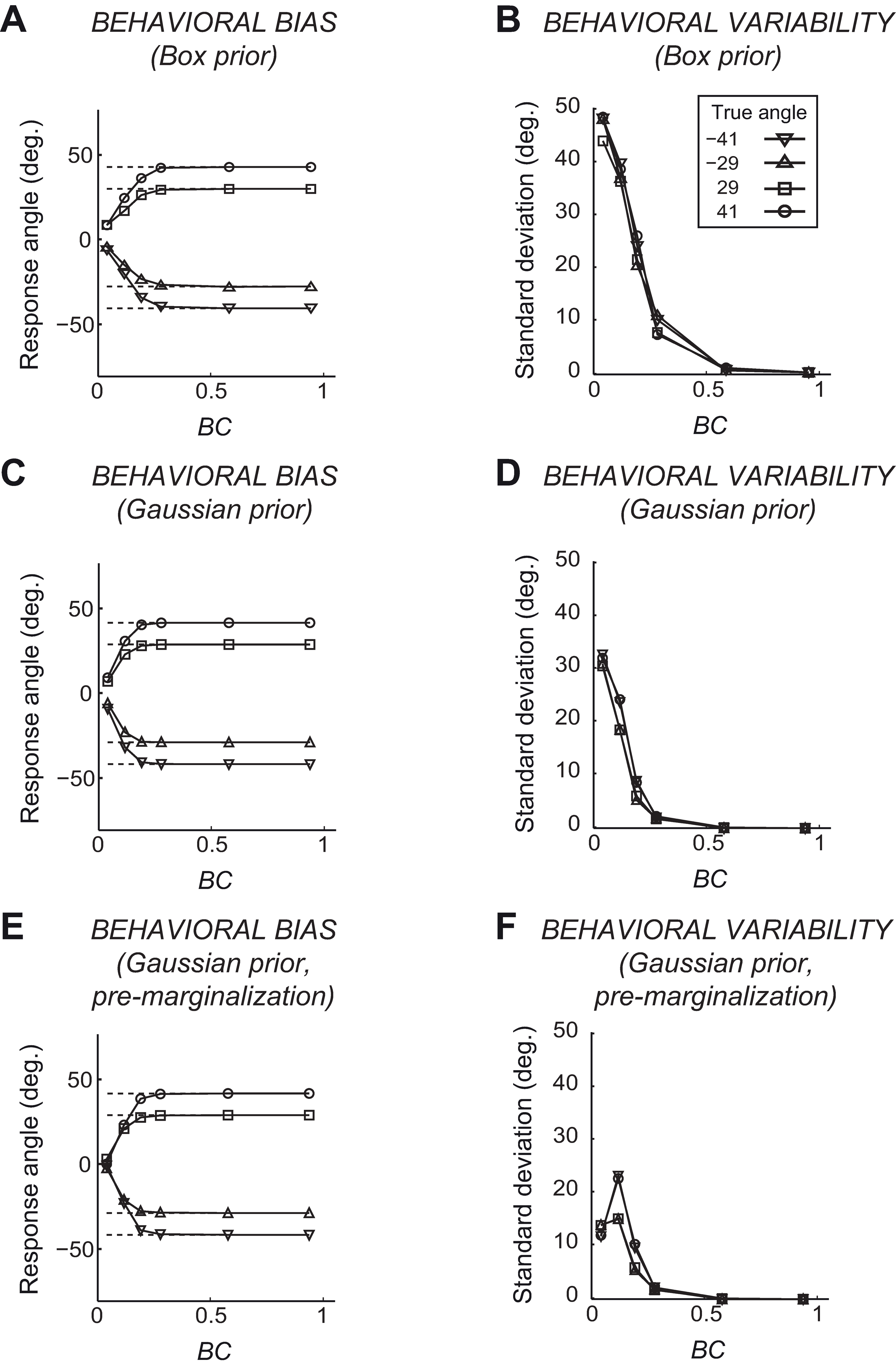

Supplement: S1 Fig — (A,B) mean and standard deviation of the Maximum A Posteriori (MAP) estimate of the angle of the sound source for the post-marginalization ideal observer with a box prior (reproduced from main text Fig 2). Four different values of the true angle are shown (dashed lines). At low BC, the behavior is strongly biased towards 0, the center of the prior belief of the observer, whereas at high BC it correctly recovers the correct value. (C,D) behavior of the post-marginalization ideal observer when the box prior is replaced with the Gaussian prior of Fischer and Pena, 2011. The mean behavior is almost identical, while the standard deviation of the behavior is reduced at low values of BC (E,F) behavior of the pre-marginalization ideal observer with the Gaussian prior of Fischer and Pena 2011. At low values of BC, the pre-marginalization ideal observer mostly ignores the data, resulting in stronger bias of the mean behavior and lower standard deviation at low values of BC. At high values of BC, the post-marginalization ideal observer correctly estimates BC and both observers have very similar behavior. (TIF) [file pcbi.1008138.s010.tif]

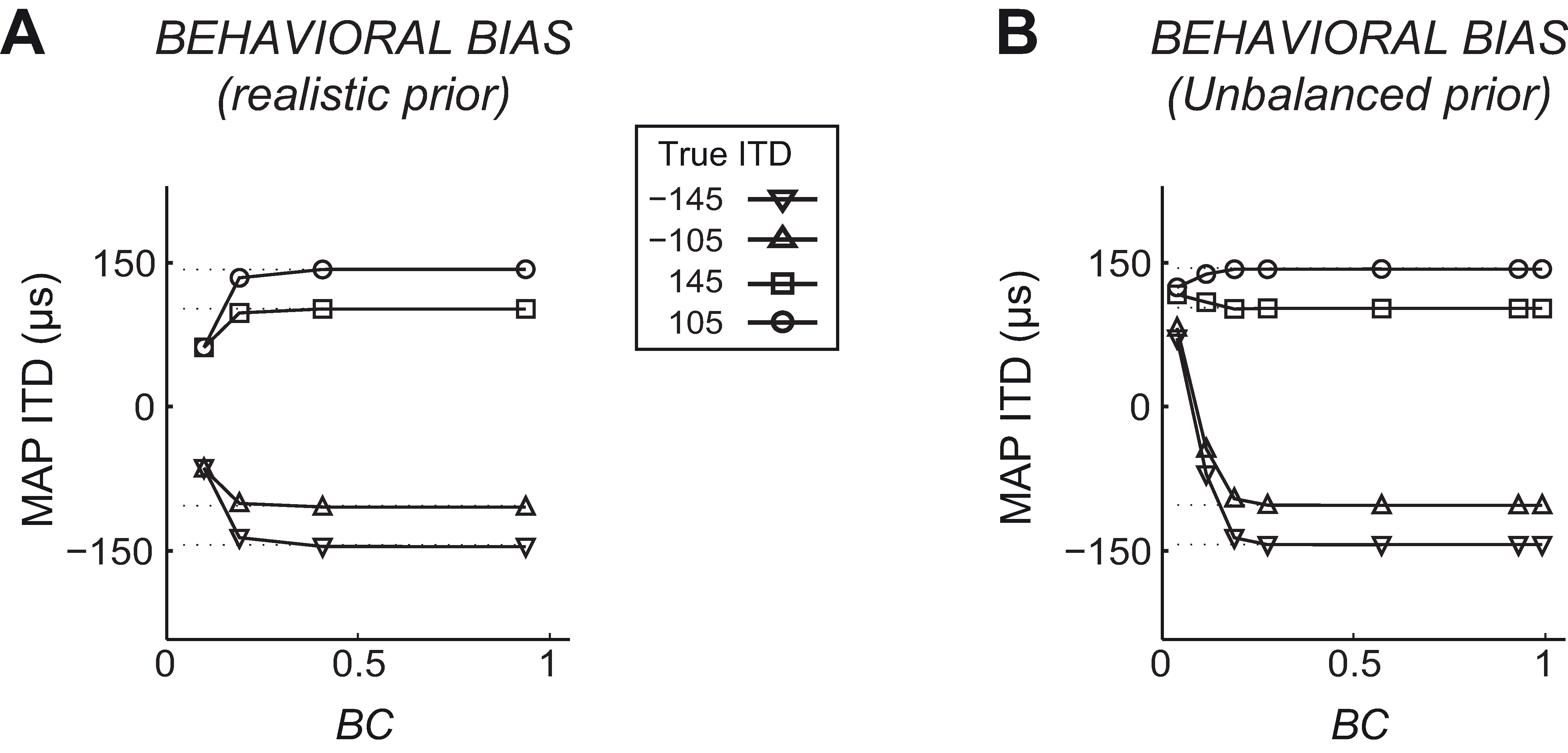

Supplement: S2 Fig — (A,B) The mean behavior of the Bayesian ideal observer (post-marginalization) is biased at lower values of BC. This bias is towards the center of the prior: in the realistic case, 0, (box prior over [−250μs, 250μs] range; panel A), and towards 100 for an unbalanced prior (box prior over [−250μs, 500μs]; panel B). (TIF) [file pcbi.1008138.s011.tif]

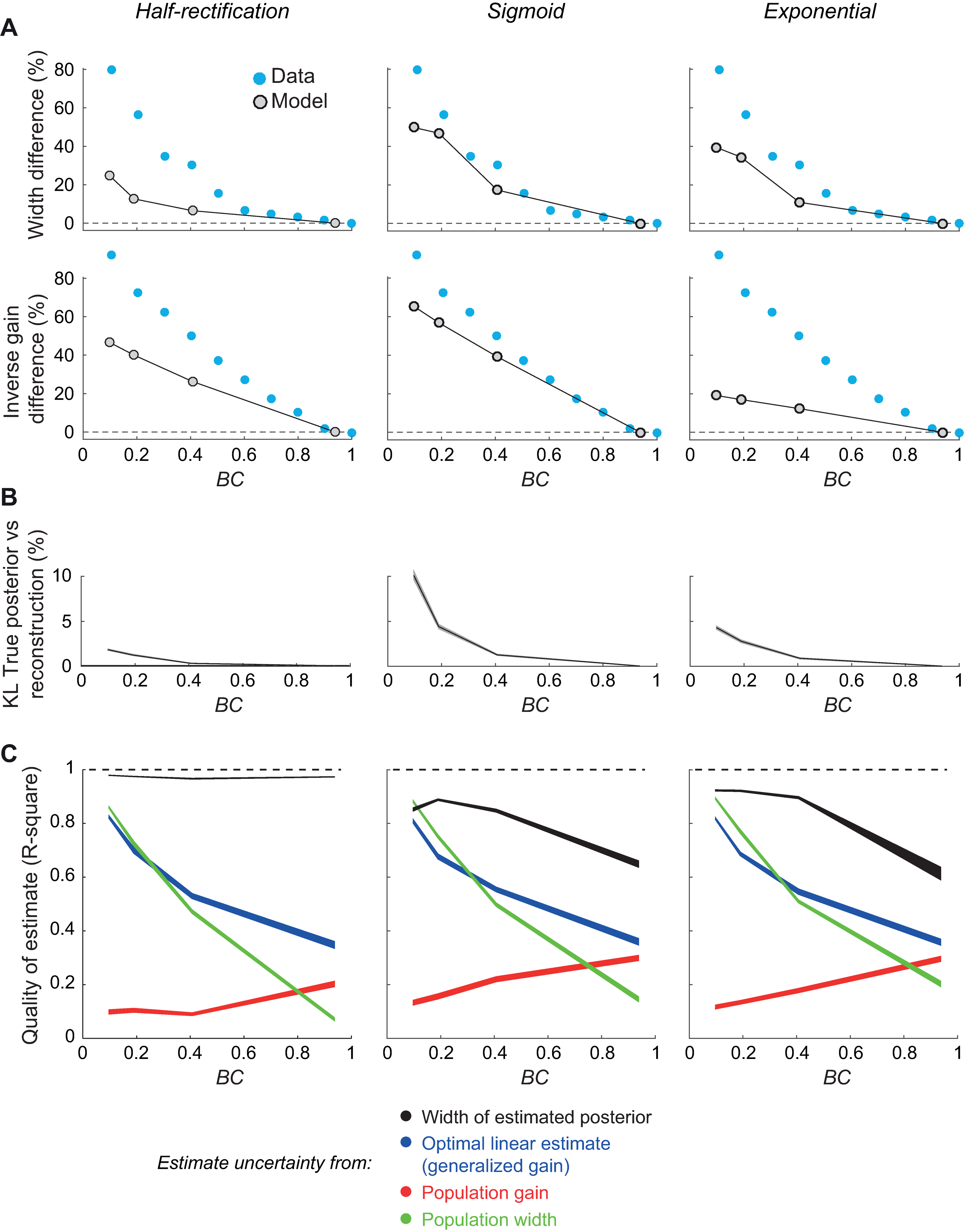

Supplement: S3 Fig — Each column correspond to a different model, with the static nonlinearity indicated at the top. (A) Width (top row) and inverse gain (bottom) of the tuning curves, as a function of BC, expressed as a percent change from the value measured at the highest BC. Gray symbols: models. Blue: data from Cazettes et al.. (B) Average KL divergence between the posterior distribution computed by the ideal observer and the posterior decoded from the model population activity. Same conventions as in Fig 3B. (C) Performance of different estimators of uncertainty. Same conventions as in Fig 5A. Notice how, even in the sigmoid model in which there are large changes of width across different values of BC, the width gives poor reconstructions of the uncertainty. (TIF) [file pcbi.1008138.s012.tif]

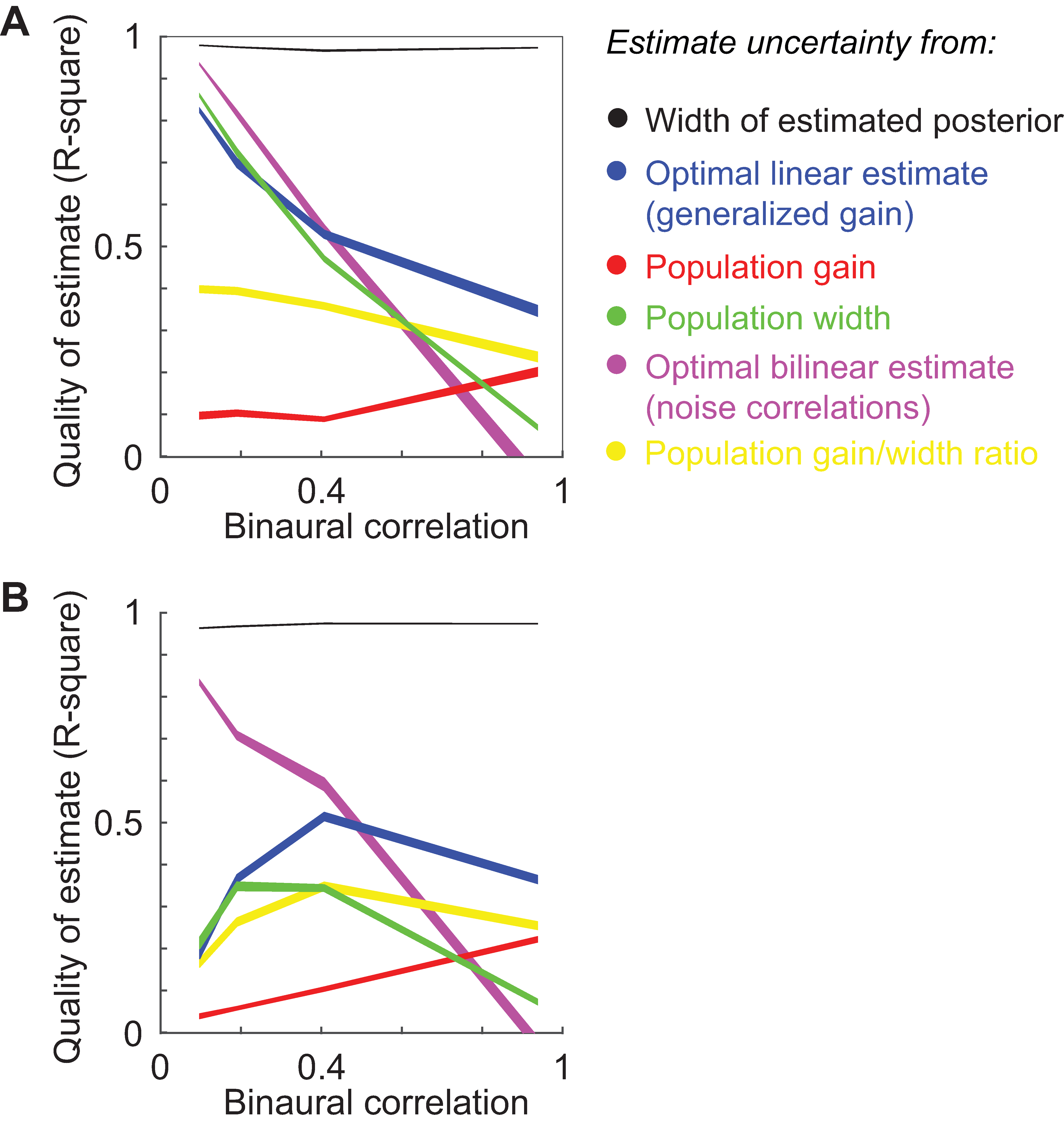

Supplement: S4 Fig — A. Comparison in the auditory model (as in Fig 3D), with two additional features: all products of neuronal activity (as a surrogate of neuronal correlations), and the gain/width ratio of the neuronal activity. Uncertainty is defined as the log-variance as in the main text. The optimal bilinear estimate overfits at the largest value of BC thus leading to a negative R-square value. B. Same as A but uncertainty is defined as the posterior log-entropy instead. (TIF) [file pcbi.1008138.s013.tif]

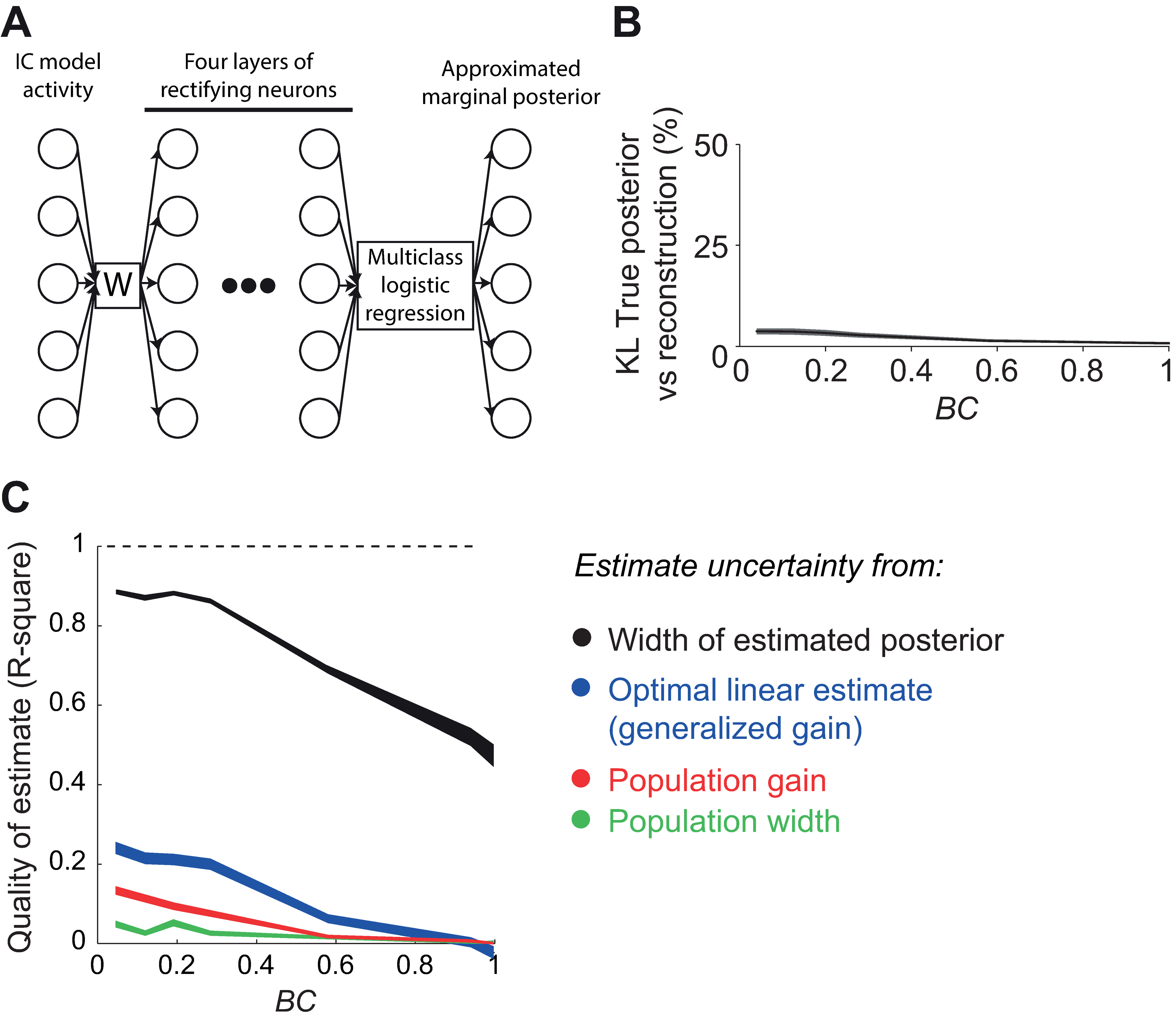

Supplement: S5 Fig — (A) Schematic of the deep network architecture. The marginal posterior is approximated from the IC model activity by four layers of neurons implementing a rectifying nonlinearity. The weight matrices W are trained to achieve the best approximation. (B) Average KL divergence between the posterior distribution computed by the ideal observer and the posterior decoded from the model population activity. Same conventions as in Fig 3B. (C) Performance of different estimators of uncertainty. Same conventions as in Fig 5. (TIF) [file pcbi.1008138.s014.tif]
